# Yeast Ppz1 protein phosphatase toxicity involves the alteration of multiple cellular targets

**DOI:** 10.1038/s41598-020-72391-y

**Published:** 2020-09-24

**Authors:** Diego Velázquez, Marcel Albacar, Chunyi Zhang, Carlos Calafí, María López-Malo, Javier Torres-Torronteras, Ramón Martí, Sergey I. Kovalchuk, Benoit Pinson, Ole N. Jensen, Bertrand Daignan-Fornier, Antonio Casamayor, Joaquín Ariño

**Affiliations:** 1 Institut de Biotecnologia i Biomedicina & Departament de Bioquímica i Biologia Molecular, Universitat Autònoma de Barcelona, Cerdanyola del Vallès, Spain; 2 Research Group on Neuromuscular and Mitochondrial Diseases, Vall d’Hebron Research Institute, Universitat Autònoma de Barcelona, Barcelona, and Biomedical Network Research Centre on Rare Diseases (CIBERER), Instituto de Salud Carlos III, Barcelona, Spain; 3 Department of Biochemistry & Molecular Biology and VILLUM Center for Bioanalytical Sciences, University of Southern Denmark, Odense, Denmark; 4 Bordeaux University, IBGC CNRS UMR 5095, Bordeaux, France; 5 Service Analyses Metaboliques TBMcore CNRS UMS3427/INSERM US05, Université de Bordeaux, Bordeaux, France; 6 Present Address: Institute of Bioengineering, School of Engineering, Ecole Polytechnique Federale de Lausanne, Lausanne, Switzerland; 7 Present Address: Laboratory of Bioinformatic Approaches in Combinatorial Chemistry and Biology, Department of Functioning of Living Systems, Shemyakin-Ovchinnikov Institute of Bioorganic Chemistry, Moscow, Russia

**Keywords:** Biochemistry, Genetics, Microbiology, Molecular biology

## Abstract

Control of the protein phosphorylation status is a major mechanism for regulation of cellular processes, and its alteration often lead to functional disorders. Ppz1, a protein phosphatase only found in fungi, is the most toxic protein when overexpressed in *Saccharomyces cerevisiae*. To investigate the molecular basis of this phenomenon, we carried out combined genome-wide transcriptomic and phosphoproteomic analyses. We have found that Ppz1 overexpression causes major changes in gene expression, affecting ~ 20% of the genome, together with oxidative stress and increase in total adenylate pools. Concurrently, we observe changes in the phosphorylation pattern of near 400 proteins (mainly dephosphorylated), including many proteins involved in mitotic cell cycle and bud emergence, rapid dephosphorylation of Snf1 and its downstream transcription factor Mig1, and phosphorylation of Hog1 and its downstream transcription factor Sko1. Deletion of *HOG1* attenuates the growth defect of Ppz1-overexpressing cells, while that of *SKO1* aggravates it. Our results demonstrate that Ppz1 overexpression has a widespread impact in the yeast cells and reveals new aspects of the regulation of the cell cycle.

## Introduction

In *Saccharomyces cerevisiae*, the type 1-related Ser/Thr protein phosphatase Ppz1 is a 692-residue protein composed of a C-terminal catalytic domain and a long N-terminal segment (≈ 350 residues). The C-terminal domain is ≈ 60% identical to Glc7, the yeast PP1 catalytic subunit (PP1c), whereas the N-terminal region is unrelated to other yeast polypeptides. *PPZ1* has a paralog, *PPZ2*, whose product is highly similar to Ppz1 at the catalytic domain (86% identity), bur far less conserved (43% identity) at the N-terminal extension^1–3^. In contrast to Glc7, which represents the ubiquitous PP1c, PPZ enzymes are restricted to fungi^4,5^.

Ppz1 has an important role in monovalent cation homeostasis, and deletion of *PPZ1* results in increased salt tolerance^6^. This effect has been attributed to a role of Ppz1 in two different cellular processes: (i) the negative control of K^+^ uptake through the high-affinity Trk transporters, and ii) a repressive effect on expression of the *ENA1* gene, encoding a Na^+^/K^+^-ATPase involved in the response to salt stress^6–9^. The increased turgor pressure due to augmented K^+^ influx in *ppz1* cells was proposed to explain the sensitivity of this strain to alterations in the integrity of the cell wall^3,6,10,11^. Recent work has linked Ppz1 with the dephosphorylation of ubiquitin Ser-57 and, therefore, with the regulation of endocytic trafficking and ubiquitin turnover^12^. In addition, Ppz1-mediated dephosphorylation of Art1 has been found necessary for interaction of this arrestin with Mup1 in response to the presence of methionine^13^. Ppz1 has been identified as a virulence factor in some important human pathogenic fungi, such as *Candida albicans*^14^ and *Aspergillus fumigatus*^15^, although it has been recently found that this is not a common trait for all fungal pathogens^16,17^.

Ppz1 is regulated in vivo by Hal3 and Vhs3, which act as inhibitory subunits. Both subunits bind to the catalytic domain of the phosphatase^10,18,19^, although Hal3 is the more relevant inhibitor in vivo*.* Remarkably, in *S. cerevisiae* and related fungi, Hal3 and Vhs3 are moonlighting proteins. In addition to Ppz1 inhibition, they associate with Cab3, forming an unusual heterotrimeric phosphopantothenoylcysteine decarboxylase (PPCDC) enzyme^20–22^, which catalyzes a fundamental step in the biosynthesis of coenzyme A (CoA). Consequently, a double *hal3 vhs3* mutant is not viable because of the inability to synthetize CoA^19,20^.

Deregulation of phospho-dephosphorylation processes often leads to cell abnormalities or even cell death, and is at the basis of many human diseases^23^. The need for fine regulation of Ppz1 function was first deduced from the observation that expression of the phosphatase from an episomal plasmid, driven either by the native *PPZ1* promoter or the strong inducible *GAL1-10* promoter, severely reduced or even blocked cell proliferation^10,24^. These effects were counteracted in full or in part by high dosage of the *HAL3* gene. More recently, a genome-wide study to find dosage-sensitive genes, based on a genetic tug-of-war (gTOW) strategy, identified *PPZ1* as the gene for which the cell has the lowest tolerance limit^25^, indicating that Ppz1 is the most toxic protein when overexpressed in budding yeast. Earlier studies revealed that overexpression of Ppz1 blocks the cell cycle at the G_1_/S transition step and is accompanied with a delay in the expression of the G_1_ phase cyclins Cln2 and Clb5. Very recent work in our laboratory has proved that the toxic effect of the excess of Ppz1 is indeed due to the increase in phosphatase activity, ruling out the possibility that it might be caused by down-titration of the necessary Hal3 and Vhs3 components of the PPCDC enzyme^26^. Interestingly, Ppz1-overexpressing cells do not die, but remain blocked at G_1_ phase. In addition, we have stablished that Ppz1 can be detected associated with polysomes and that the excess of the phosphatase likely impairs protein translation, resulting in a Gcn2-mediated increase in the phosphorylation of the translation initiation factor eIF2α^26^.

However, beside these evidences, the reasons for the toxicity of higher-than-normal levels of Ppz1 activity are still obscure. In this work, we have approached this question by defining the changes in the transcriptome and the phosphoproteome in cells overexpressing Ppz1. We have detected changes in the mRNA levels of more than one thousand genes and identified changes in over 400 phosphosites, mostly dephosphorylation events, during the first four hours of Ppz1 overexpression. Our findings provide clues to understand the molecular basis of Ppz1 toxicity, which would involve appearance of oxidative stress and possible DNA damage, alteration of purine nucleotide metabolism, and disruption of key signaling pathways, including the rapid dephosphorylation of Snf1 and its downstream transcription factor Mig1, and the phosphorylation of Hog1 and its downstream transcription factor Sko1.

## Results

### Changes in transcriptomic profile induced by overexpression of Ppz1

**Figure 1:**
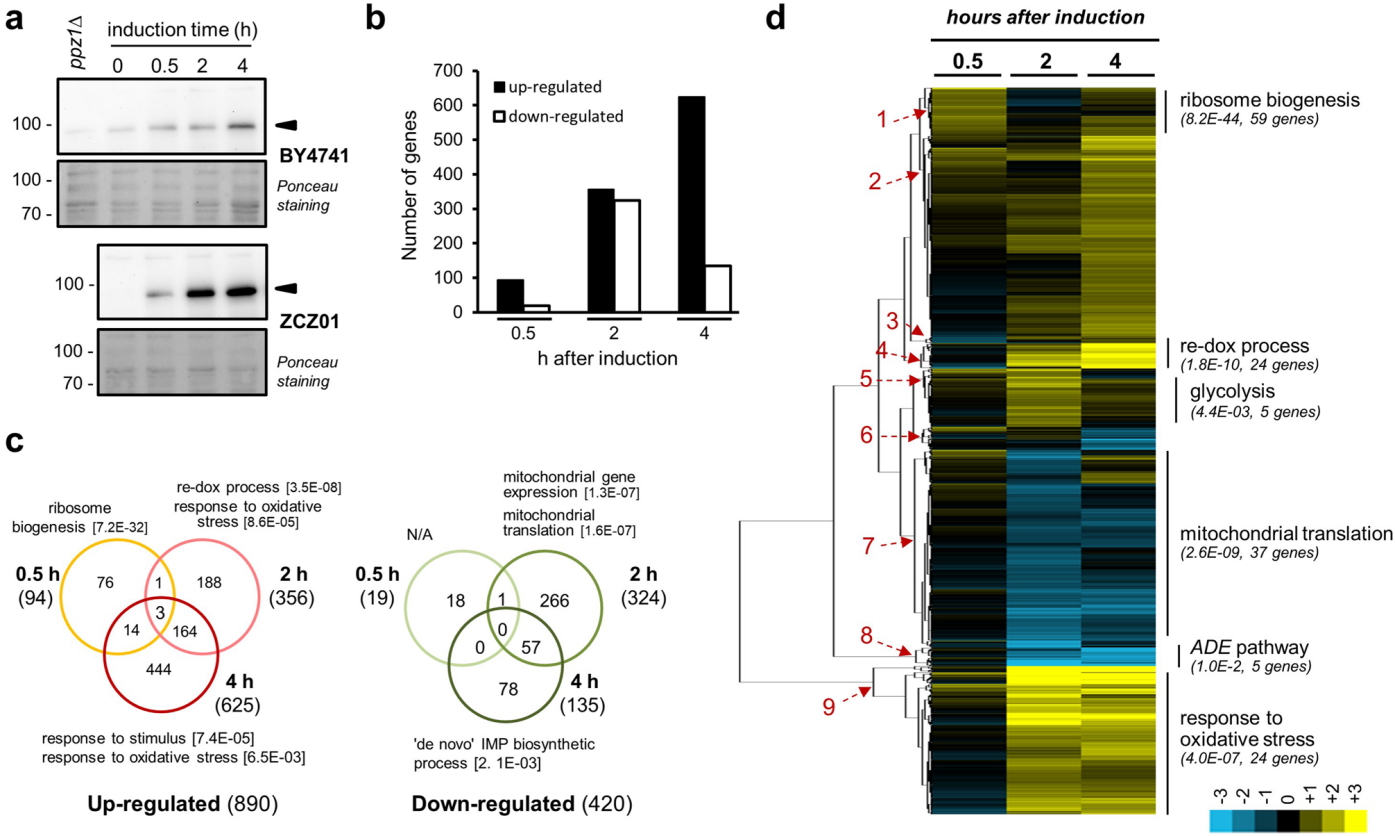
Changes in transcriptomic profile upon overexpression of *PPZ1*. (**a**) Wild-type BY4741 and its isogenic derivative ZCZ01 (*GAL1-10:PPZ1*) cells were grown on YP with 2% raffinose (YP-Raff) until OD_600_ = 0.5 and then 2% galactose was added. Samples were taken at the indicated times, electrophoresed (40 μg of protein) and immunoblotted using polyclonal anti-Ppz1 antibodies. Extract from a *ppz1*Δ deletant is included as negative control. Arrowheads denote Ppz1 signal. Ponceau staining is shown for loading and transfer reference. All samples were loaded in the same gel and are shown separated for illustrative purposes. (**b**) Time-course distribution of up- and down-regulated genes. (**c**) Venn diagrams showing the number of genes whose expression was induced or repressed (≥ twofold, *p* < 0.05) at different times upon overexpression of *PPZ1*. Total numbers of genes in each category are in parentheses. Gene Ontology annotations, generated by YeastMine at SGD (https://yeastmine.yeastgenome.org/) with default settings, are also shown (*p*-values are in brackets). (**d**) Expression changes for 1,294 genes showing induction or repression at least at one time-point (mean values) were subjected to hierarchic clustering (Euclidean distance/complete linkage) using the Gene Cluster software v. 3.0^27^. The result was visualized with Java Tree View v. 1.145^28^. Nine major cluster were obtained and the specific GO enrichment for several of them, including *p*-value and number of genes belonging to the specific category, is shown in parentheses. The intensity of the expression change can be inferred by comparison with the enclosed scale (log(2) values).

To evaluate the impact of high levels of Ppz1 on genome-wide mRNA levels, wild type BY4741 strain and its derivative ZCZ01, expressing *PPZ1* from the *GAL1-10* promoter, were grown on raffinose and *PPZ1* expression induced by addition of galactose. Total RNA was prepared from samples taken at 30 min, 2 h and 4 h upon induction and subjected to RNA-seq. These time-points cover from the early onset of Ppz1 appearance to the establishment of high steady-state levels of the phosphatase and correspond to significant delay (2 h) and full blockage of cell cycle (4 h) (see Fig. 1A and reference^26^). Data from three experiments was combined and evaluated. Altogether 890 genes were induced and 420 were repressed at least at one time-point (Supplementary Table S1). Analysis of the changes as a function of time showed (Fig. 1B) that the expression of 94 genes was induced at least twofold after 30 min of addition of galactose, whereas only 19 were down-regulated. Induced genes increased up to 356 after 2 h and even further (625) at 4 h. In contrast, the peak of repressed genes was found at 2 h (324) and decreased to 135 at 4 h (Fig. 1B). Among them, 16 were induced at least at one time-point and repressed at another time-point.

Gene Ontology analysis revealed specific traits for gene expression along the experiment. As shown in Fig. 1C, early response (0.5 h) was characterized by a moderate but very consistent increase in the expression of more than 50 genes related to ribosome biogenesis (*p* < 7.2E−32). Further analysis revealed that most of these genes were involved rRNA processing (*p* < 3.7E−25). The pattern drastically changed at 2 and 4 h, when many genes related to the response to oxidative stress emerged (such as *GRE1*, *CTT1*, *TSA2*, *SRX1*, *AHP1*, *GPX1* or *GTT2*). Analysis of the set of repressed genes did not show any specific enrichment after 0.5 h. However, the list of repressed genes at 2 h was enriched in genes required for mitochondrial translation, most of them encoding proteins that are components of both the large and small ribosomal subunits. At this time, it was also observed repression of diverse genes required for effective G_1_ to S phase cell cycle transition or known to peak at G_1_, such as *CLN2*, *CLB5*, *PCL1*, *SWI4*, *CDC21* or *TOS4*, as well as of several protein kinases involved in septin assembly and checkpoint (*KCC4, GIN4, HSL1*). The profile changed after 4 h and, in this case, we found repressed many of the genes involved in the synthesis of IMP from 5-phosphoribosyl-1-pyrophosphate (PRPP), such as *ADE8*, *ADE2*, *ADE1*, *ADE13*, and *ADE17* (these last four genes responsible for the last five reactions of the pathway). In fact, examination of the entire pathway (see below) revealed that other genes, such as *ADE4*, *ADE5*,7 and *ADE6* were also repressed (they were not included in the initial analysis because their *p*-value was around 0.1, above the *p* < 0.05 threshold). In fact, *ADE12*, necessary for the transformation of IMP into AMP was also repressed. Therefore, the entire pathway from PRPP to AMP appears downregulated upon overexpression of *PPZ1*. An overall view of the expression changes is presented in Fig. 1D as a heat-map generated from the results of gene clustering according to their expression profile. Note that among the nine major clusters generated, all but clusters 2 and 6 are enriched in genes that are functionally related.

Transcriptomic data was mapped onto the YeastPathways database of *S. cerevisiae* metabolic pathways and enzymes, and the Pathway Perturbation Score (PPS) was calculated. PPS is the measure of the overall extent to which a pathway is up- or down-regulated, by combining the activation levels of all reactions in the pathway^29^. As shown in Supplementary Fig. S1A, the three top scores were β-alanine biosynthesis, superoxide radical degradation (both of them activated), and IMP biosynthesis (downregulated).

The response of three different promoters from genes that were induced (*GAP1*, *GRE2*, and *NCE103*) plus one gene that was repressed (*SIT1*) was further tested using *LacZ* reporters. As shown in Supplementary Fig. S1B, the response for all four genes was confirmed. The observed changes were relatively moderate (1.5 to threefold), likely due to the fact that overexpression of Ppz1 inhibits protein translation^26^.

### Ppz1 overexpression triggers ROS accumulation and possibly DNA damage

**Figure 2:**
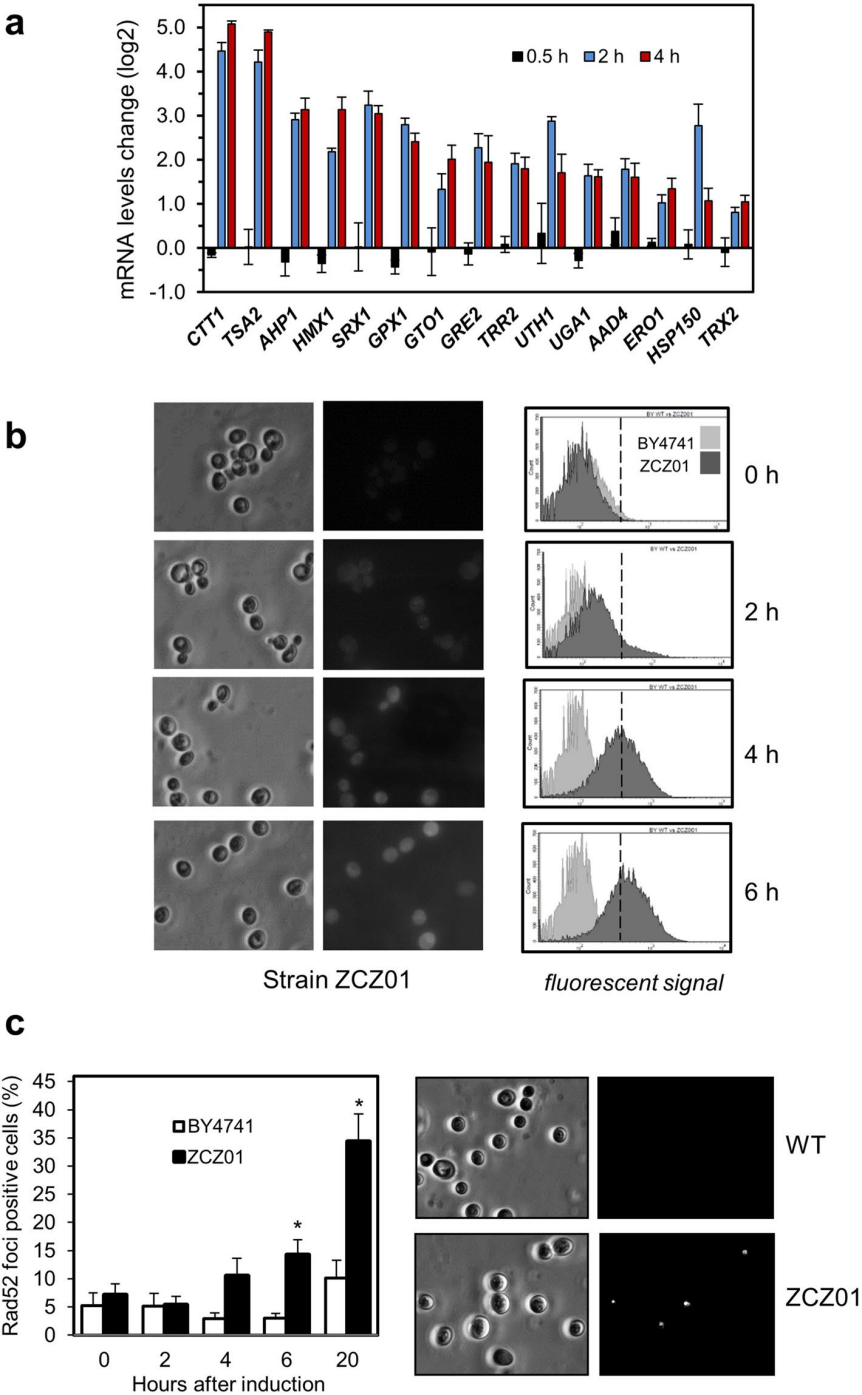
Overexpression of Ppz1 causes oxidative stress and signals through Rad52. (**a**) Changes in mRNA levels for selected genes calculated from RNA-seq data (log2 of ZCZ01/WT ratio). Values are mean ± SEM from 3 experiments. (**b**) Cells grown on YP raffinose received 2% galactose to induce expression of Ppz1 and, at the same time, were loaded with dihydrorhodamine 123. Samples were taken at the indicated times and subjected to fluorescence microscopy (left panel) or processed for flow cytometry (right panel). The presence of ROS oxidizes dihydrorhodamine 123 to the fluorescent derivative rhodamine 123. Only the ZCZ01 strain is shown since wild type BY4741 cells produced no fluorescence at any time tested. **c**) The indicated strains were transformed with plasmid pWJ1344, which expressed an YFP-tagged version of Rad52. Upon growth on 2% raffinose, cells were shifted to 2% galactose. Samples were taken at different times and the percentage of cells positive for Rad52 foci was estimated. Data is presented as the mean ± SEM from four experiments (average 160–430 counted cells per experiment and time point). A representative micrograph taken after 20 h is shown on the right.

The transcriptomic profile described above prompted us to test the possibility that high levels of Ppz1 might trigger oxidative stress. As shown in Fig. 2A, diverse genes known to be transcriptionally induced in response to oxidative stress^30–33^ are consistently up-regulated in a time-dependent manner by overexpression of Ppz1. We found that such induction is accompanied by the presence of ROS, as determined by incubation of Ppz1-overexpressing cells with the dye dihydrorhodamine 123, which becomes fluorescent upon oxidation. Fluorescence could be detected by both microscopic observation and flow cytometry (Fig. 2B). A well-known effect of oxidative stress is the occurrence of DNA damage. Repair of such damage often requires the recruitment of several proteins, including the endonuclease Rad52. Indeed, overexpression of Ppz1 resulted in the accumulation of cells showing Rad52 foci (Fig. 2C). Cells positive for Rad52 foci were already detected after 4 h of induction and greatly increased in number after 20 h.

### Repression of the ADE pathway in Ppz1-overexpressing cells is not caused by oxidative stress but by abnormal accumulation of ATP

**Figure 3:**
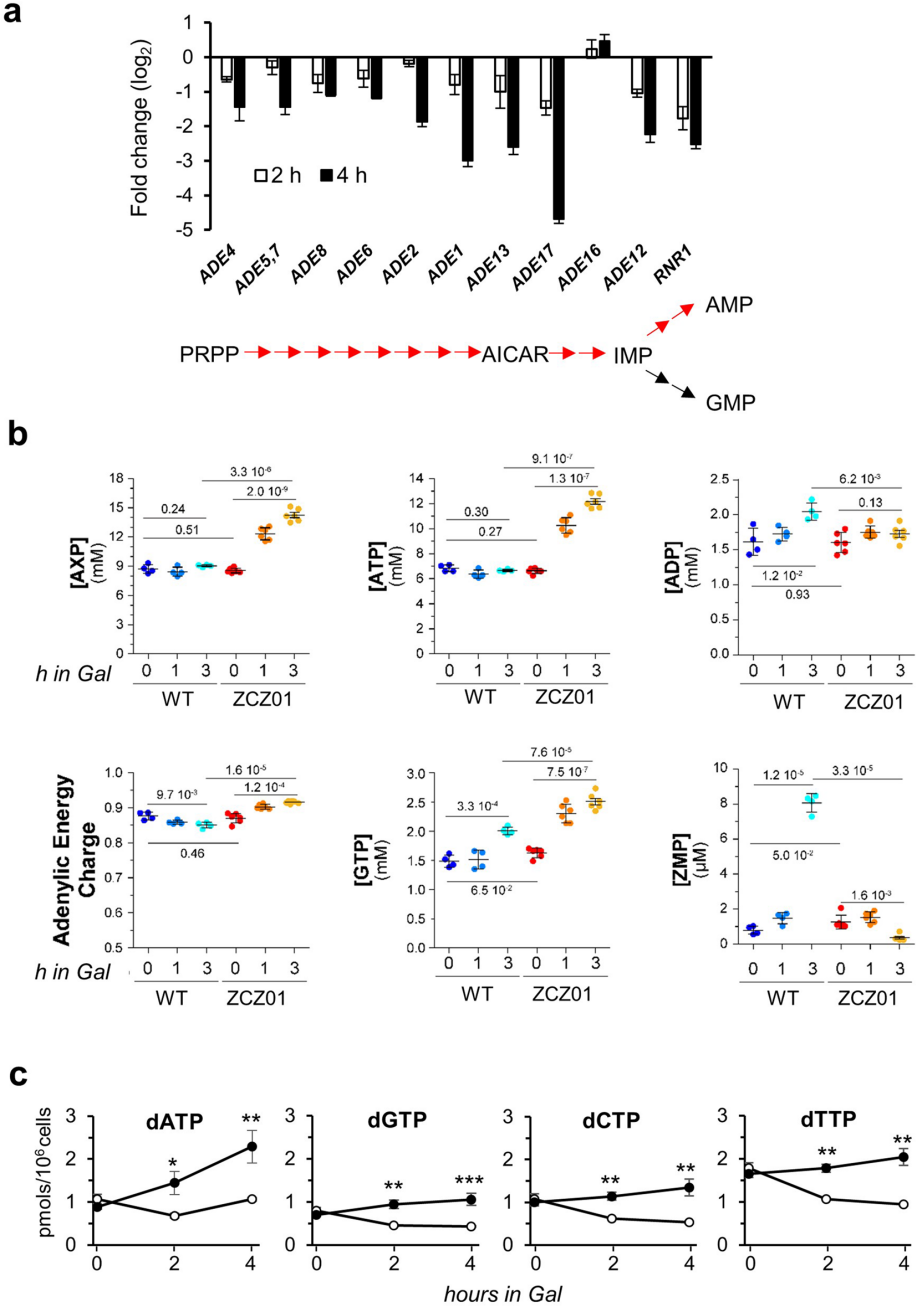
Overexpression of Ppz1 increases energetically charged purine nucleotide levels. (**a**) Expression levels of genes that define the de novo purine biosynthetic pathway derived from the RNA-seq data (ordered according to their sequence in the pathway, schematically shown at the bottom of the histogram). *RNR1*, encoding the large subunit of ribonucleotide reductase, is also included. (**b**) The intracellular concentrations of the indicated purine-containing molecules were determined in exponentially growing cells in YP-Raff (2%) with addition of galactose 2% (Gal) at time 0. Metabolic extracts were normalized to cell number and median cell volume and results (from 4 to 6 independent extractions) are given as internal concentrations ± SD of adenylic nucleotides inferred from standard curves using pure compounds. Statistical analysis was performed with Welch’s unpaired t-test (*p*-values are indicated in the graphs). The concentration of AXP is the sum of the concentrations of ATP, ADP, and AMP. ZMP (AICAR monosphosphate) is the 5-aminoimidazole-4-carboxamide ribonucleotide intermediate. (**c**) Determination of dNTPs levels in wild type (open circle, BY4741), and Ppz1-overexpressing (filled circle, ZCZ01) cells. Cells grown on YP-Raff were collected at time 0 and 2 and 4 h after addition of 2% galactose and processed as described in Material and Methods. Data correspond to the mean ± SEM from 3 to 5 independent experiments. *p < 0.05; **p < 0.01; ***p < 0.001, calculated by the Student’s t-test.

As illustrated in Fig. 3A, most genes involved in the *ADE* pathway were repressed after 2 and 4 h of Ppz1 overexpression. The vast majority of them are under the control of the Bas1 and Bas2 (Pho2) transcription factors, and previous reports had revealed that oxidative stress results in downregulation of the *ADE* pathway due to interference with the Bas1/Bas2 interaction^34^. Therefore, we hypothesized that the oxidative stress generated upon Ppz1 overexpression could be responsible for a Bas1/Bas2-mediated defect in expression of the *ADE* genes, leading perhaps to deficient de novo synthesis of adenine nucleotides and consequent cell cycle blockage. However, we observed that neither addition of adenine to the medium, nor transformation with plasmid B273, which allows expression of a Bas1/Bas2 chimera that permits constitutive expression of most of *ADE* genes, improved growth of Ppz1-overexpressing cells.

We then characterized the nucleotide profile of cells overexpressing Ppz1 in comparison with control cells (Fig. 3B). Surprisingly, our results revealed a time-dependent accumulation of adenine nucleotides ([AXP] = [ATP] + [ADP] + [AMP]) which nearly doubled the initial levels (Fig. 3B). Such change was mostly due to an increase of about twofold in ATP levels. Consequently, the adenylic energy charge (measured as [ATP] + 1/2 [ADP])/[AXP]) raised from 0.849 ± 0.008 to 0.915 ± 0.004 (*p* < 1.6E-5). GTP levels also increased significantly in about 25%. In addition, a clear-cut increase in NAD^+^ levels of about twofold was observed (not shown). Most of these changes were already significant after one hour of Ppz1 induction. In contrast, ZMP (AICAR monophosphate) levels largely increased in the wild type strain after 3 h of galactose addition, but they were almost undetectable in Ppz1-overexpressing cells (Fig. 3B). These results indicate that, in contrast to our initial hypothesis, overexpression of Ppz1 results in accumulation of purine nucleotides, essentially in the form of ATP, and in abrupt decrease in the levels of the pathway intermediate ZMP. In line with these findings, the levels of dNTPs were higher (2.1 to 2.5-fold) in cells overexpressing Ppz1 (Fig. 3C), indicating that DNA precursors were not scarce.

### Overexpression of Ppz1 provokes numerous changes in protein phosphorylation profiles

For proteomic and phosphoproteomic studies, wild type BY4741 and ZCZ01 cells were shifted to a medium containing galactose and samples taken after 30, 60, 120, and 240 min. Taking into account the kinetics of Ppz1 expression, our expectation was that changes identified at short times (30–60 min) could be directly due to Ppz1, whereas alterations occurring at longer times could be due to indirect or secondary effects. Analysis of the proteome allowed the identification of 4,041 proteins (Supplementary Table S2). Changes in abundance were very limited and relatively modest: only 10 proteins were found to increase at least twofold (Yro2, Ser3, Ssa4, Nqm1, Gnd2, Rtc3, Rse1, Ald3, Ygp1, and Ypr1). This was not surprising, as it can be explained by the recently described negative effect of overexpression of Ppz1 on protein translation^26^. All of them, with the only exception of Rtc3, also displayed consistent increases in expression at the mRNA level (Supplementary Table S3). Gene Ontology analysis revealed in this set a slight predominance (2.63E−4) of genes encoding oxido-reductases acting on alcohol groups (Ser3, Gnd2 and Ypr1).

Analysis of phosphoproteomic data provided a total number of 8,701 phosphopeptides identified in at least one experiment for any of the strains and conditions tested, corresponding to 6,008 unique phosphorylated sites (localization score ≥ 0.7, FLR < 4%^35^). Further analysis yielded 5,705 unique phosphopeptides corresponding to 1,435 different proteins. Evaluation of changes in the phosphorylation level yielded 304 unique sites with at least twofold consistent decrease (*p* < 0.05) in phosphorylation in cells overexpressing Ppz1 (82,6% S; 15.8%, T; 1.6%, Y), corresponding to 134 different proteins (Supplementary Table S4). On the other hand, only 80 phosphosites (71.2% S, 27.5%, T; 1.3%, Y), corresponding to 36 different proteins, showed at least twofold increase in phosphorylation (Supplementary Table S4).

**Figure 4:**
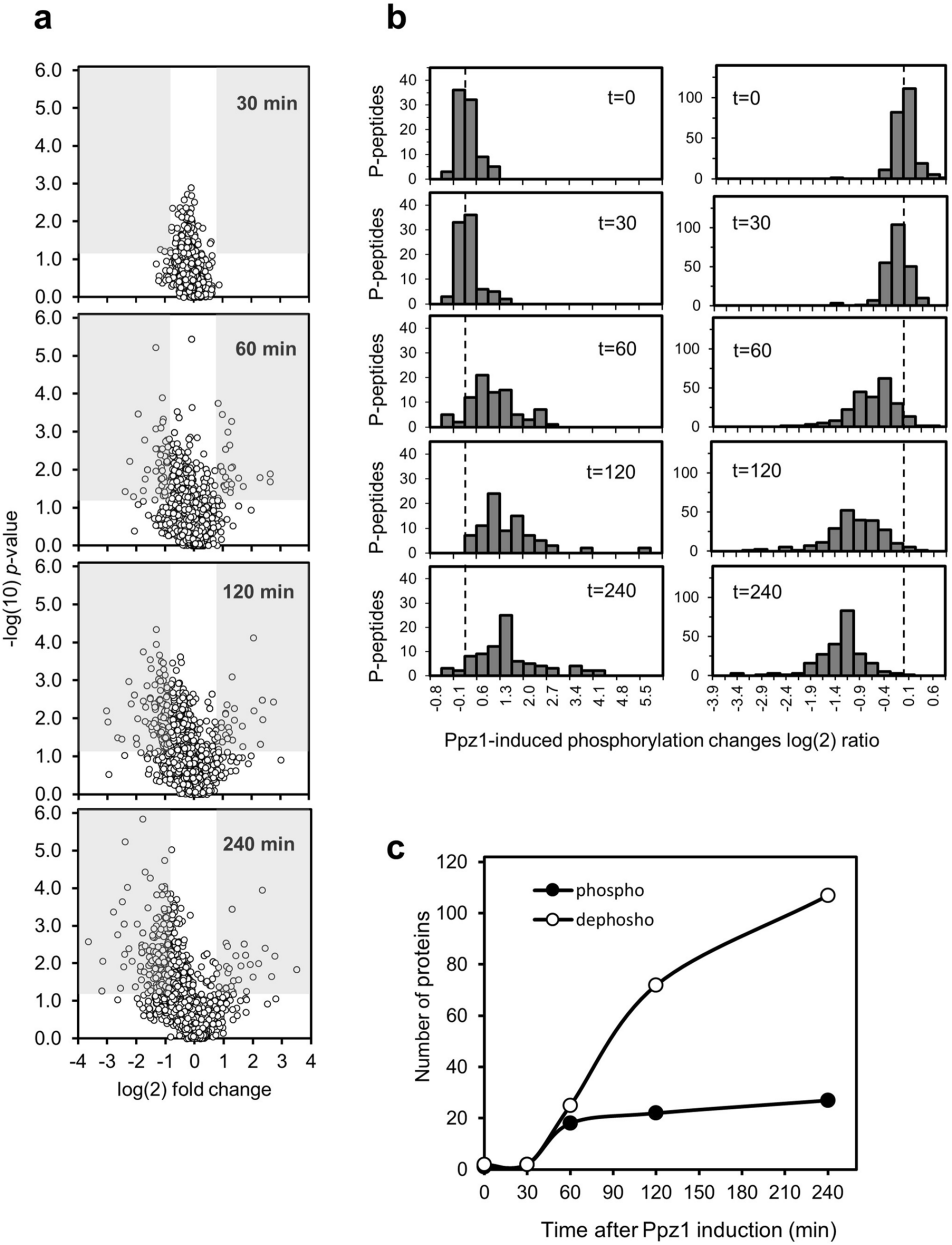
Changes with time in phosphorylation profile upon overexpression of Ppz1. (**a**) The significance versus fold-change in phosphorylation (y and x axes, respectively) of the 6,008 unique phosphosites identified in this work and their variation along time with overexpression of Ppz1 is represented as a Volcano plot. Fold-change is calculated as the ratio of Ppz1-overexpressing cells *vs* control cells, for each time and compared with t = 0. Shadows denote regions in which log(2) value of the change is ≥ 1 (phosphorylated) or ≤ − 1 (dephosphorylated), and p-value ≤ 0.05. (**b**) Data for a total number of 230 dephosphorylated (right panel) and 85 phosphorylated phosphopeptides (left panel) showing statistically significant changes at least at one time-point was log(2) transformed and plotted. The discontinuous line marks the zero point in the X-axis. (**c**) Variation of the number of phospho- (filled circle) and dephosphorylated (open circle) proteins along the experiment.

Examination of the kinetics of changes in phosphorylation shows that relevant changes in the phosphorylation status of the 6,008 phosphosites is only detected after 60 min of Ppz1 overexpression (Fig. 4A). A clear-cut increase in phosphorylated peptides is detected after 60 min of Ppz1 overexpression, and relatively minor changes occurs after 120 min (Fig. 4B, left panel). In contrast, a slight modification in the overall profile of dephosphorylated peptides is already seen after 30 min, becoming very evident at 60 min and showing a further shift towards dephosphorylated forms at 120 and 240 min (Fig. 4B, right panel). The representation of the number of different proteins affected in their phosphorylation state indicates that both phospho- and dephosphorylation events initiate between min 30 and 60. At 60 min the figures are not too different (18 phosphorylated and 25 dephosphorylated). However, after 60 min the number of phosphorylated proteins is stabilized, whereas that of dephosphorylated proteins increases over fourfold at 240 min (Fig. 4C). It must be noted that overexpression of Ppz1 caused dephosphorylation of certain residues and phosphorylation of others in six specific proteins: Gdc6, Rtg1, Cdc3, Seg1, Tpo3, and Ppz1 itself, which appears dephosphorylated at Thr171 at the initial stage of the experiments and becomes phosphorylated at Ser265 at 60 min.

**Figure 5:**
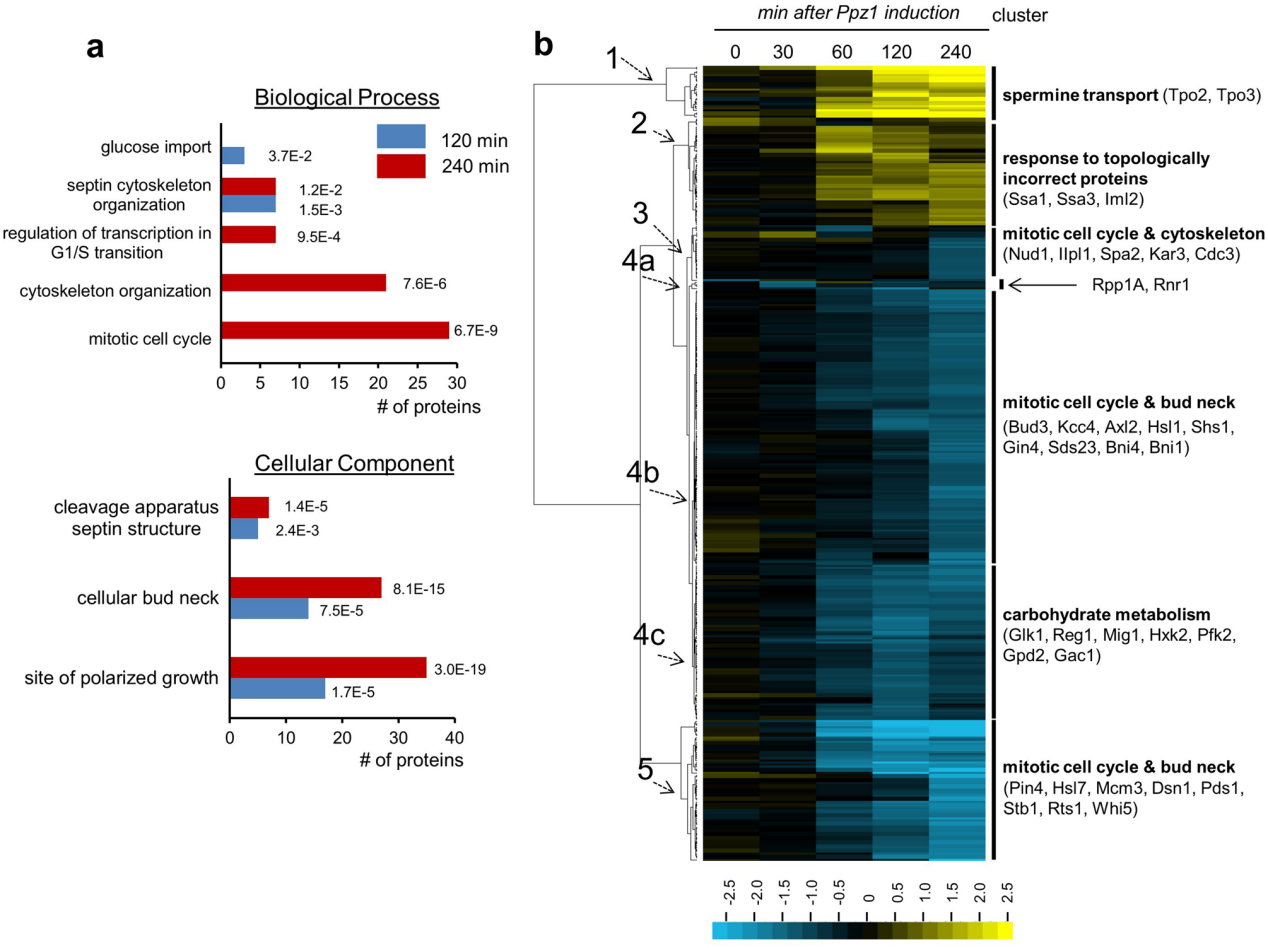
Clustering analysis of phosphoproteome changes upon Ppz1 overexpression. (**a**) The set of proteins dephosphorylated at t = 120 and t = 240 (72 and 107, respectively) were subjected to Gene Ontology analysis using the YeastMine tool at SGD with default settings. (**b**) Data from 385 phosphosites (phospho- or dephosphorylated) was calculated as the ratio between Ppz1-overexpressing cells and control cells. Log(2) transformed data was clustered with the Gene Cluster software (Euclidean distance/complete linkage) and the result was visualized with Java Tree View (v. 1.145). The intensity of the phosphorylation change can be deduced by comparison with the enclosed scale (log(2) values). Data is presented for all time-points even if in some cases the *p*-value between experiments is > 0.05.

Time-course evaluation of the dephosphorylated proteins (Supplementary Table S4) and Figs. 4C and 5A,B) revealed only two proteins with dephosphorylated sites after 30 min of Ppz1 induction: Rpp1A, the ribosomal stalk protein P1 alpha, involved in the interaction between translational elongation factors and the ribosome, and Rnr1, the major isoform of the large subunit of ribonucleotide-diphosphate reductase. After 60 min, 25 proteins resulted dephosphorylated at least at one site (49 sites in total). Five of them showed functional links with the bud neck (Hsl7, Shs1, Gnp1, Rts1 and Sec8). This tendency was intensified after 120 min of Ppz1 overexpression, when 72 proteins (142 sites) were found (Fig. 5A). Among them, 14 proteins *(p*-value 7.5E−5) were related to the bud neck (including the five dephosphorylated at 60 min), of which five (Kcc4, Shs1, Rga2, Gin4, Cdc3, and Rts1) are involved in organization of the septin ring. Interestingly, at this time all three glucose phosphorylating enzymes (Hxk1, Hxk2 and Glk1), plus the β-subunit of phosphofructokinase Pfk2, as well as Mig1 and Reg1 (the two latter, together with Hxk2, involved in the glucose repression mechanism), were also dephosphorylated. The number of dephosphorylated proteins after 240 min raised to 108 (244 sites). Twenty-nine of them (*p*-value 6.7E−09) were related to the mitotic cell cycle, of which 21 are specifically involved in cytoskeleton organization (mostly actin cytoskeleton), whereas other 7 (9.5E−04) are implicated in regulation of transcription involved in the G_1_/S cell cycle transition (Yph1, Spt6, Stb1, Whi5, Swi4, Swi5, and Swi6). Gene Ontology analysis of the total set of 134 proteins dephosphorylated at least at one time-point confirmed a strong incidence of proteins involved in mitotic cell cycle (6.19E−10) and located in sites of polarized growth (7.8E−16), particularly in the bud neck (2.3E−12).

Gene Ontology analysis of the 36 proteins in which overexpression of Ppz1 induced increased phosphorylation of at least one site showed common traits from min 60 onwards. Thus, six proteins related to the cytoplasmic ribonucleoprotein granules (Hek2, Ksp1, Rie1, Bre5, Tif4631, and Ded1) were identified. Interestingly, the last two are involved in translation initiation (Tif4631 is the translation initiation factor eIF4G, and Ded1 is an ATP-dependent DEAD-box RNA helicase required for translation). Two other translation-related proteins were also found: Sui3 and Gcd6, corresponding to the β-subunit and the catalytic ε subunit of the translation initiation factor eIF2, respectively. In addition, two spermine transporter, Tpo2 and Tpo3, showed a sustained increase in phosphorylation from 60 min onwards.

Hierarchic clustering of the data corresponding to the phosphopeptides whose phosphorylation state changed upon overexpression of Ppz1 is shown as a heat-map in Fig. 5B, together with Gene Ontology annotation of the predominant categories in the main clusters. As it can be observed, cluster 1 and 2 corresponded to proteins with hyperphosphorylated sites, whereas clusters 3, 4a, 4b, 4c, and 5 correspond to proteins dephosphorylated at least in one site. Notable, clusters 3, 4b and 5 are enriched in proteins involved in mitotic cell cycle (*p*-values 8.3E−06, 4.24E−09, and 2.3E−06, respectively). However, proteins in cluster 3 show a tendency to be functionally associated with the cytoskeleton, whereas those belonging to clusters 4b and 5 are related to sites of polarized growth and, in particular, with the bud neck (*p*-value of 7.5E−13 and 4.1E−09, respectively). Cluster 4c, in contrast, was enriched in plasma membrane proteins (*p*-value 9.4E−07) and in proteins functionally related to metabolism of carbohydrates (*p*-value 8.6E−05, including Glk1, Hxk2, Pfk2, Mig1, Reg1 and Gpd2).

A search for putative novel phosphorylation sites was conducted with the original set of 6,008 phosphosites corresponding to 1,354 different proteins. Our data was crossed with three different databases: YeastMine (through SGD, https://yeastmine.yeastgenome.org/), which contained 15,468 known phosphosites for our set of 1,354 proteins, the data base described by Bai and coworkers^36^, which holds a total number of 40,798 sites (4,148 proteins), and the BioGrid database^37^, release 3.5.171, with 42,908 entries for *S. cerevisiae* (19,981 phosphosites, 3,165 proteins). Our analysis showed that 466 sites (from 317 proteins) identified in this work were not present in any of the mentioned databases. Among them, 15 sites, corresponding to 13 different proteins, changed their phosphorylation state upon overexpression of Ppz1 (12 dephosphorylated and 3 phosphorylated, Supplementary Table S5).

### Prediction of putative kinases that could phosphorylate sites affected by Ppz1 overexpression

**Figure 6:**
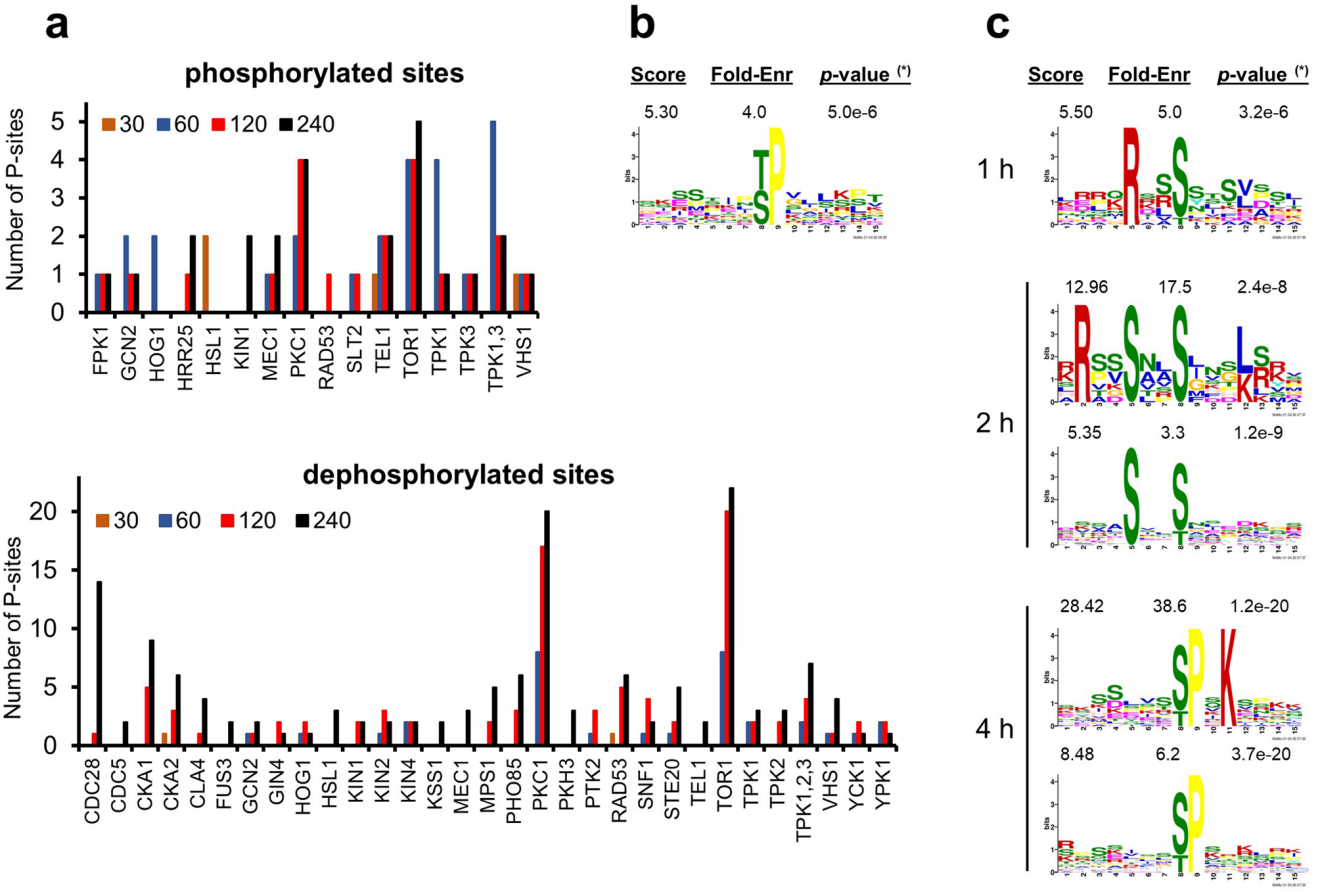
Prediction of putative kinases phosphorylating sites affected by Ppz1 overexpression. (**a**) The set of phosphosites hyperphosphorylated or dephosphorylated at any time upon overexpression of Ppz1 was submitted to the NetworKIN 3.0 high-throughput interface using the yeast Ensemble 74 database (min score = 2). If more than one kinase was assigned to a given site, only the kinase with the highest score was selected. Due to the similarity of Tpk1-3 consensus sites, the sum of all of them is also plotted. For phosphorylated sites, all kinases are shown. For dephosphorylated sites, only kinases with ≥ 2 sites are represented (the full table is available as Supplementary Table S6). (**b**) Motif enrichment using the MoMo software of phosphorylated sites, as described in Material and Methods. (*) according to the authors, the *p*-value of the Fisher Exact test on the enrichment of the motif should be interpreted as a score only. “Fold-Enr”, fold enrichment of the foreground matches *vs*. the background matches. (**c**) Time-dependent variation of motif enrichment for the dephosphorylated sites. In this case, only sites newly appearing at a given time were submitted to MoMo analysis, to better follow the variation in the motif pattern along the time of Ppz1 overexpression.

In an attempt to identify putative protein kinases responsible for the phosphorylation of sites affected by overexpression of Ppz1 we analyzed our set of phosphosites with the NetworKIN 3.0 software. Putative phosphorylating kinases could be mapped at least to one site in 26 out 36 proteins (38 sites) with increased phosphorylation, and to 82 out of 104 dephosphorylated proteins (203 sites) (Supplementary Table S6). The three top kinases related to hyperphosphorylated sites were Pkc1, Tor1 and Tpk1, although the kinetics were not identical: sites for all three kinase were identified at early times (1 h), but the number of Tpk1 sites decreased greatly after 2 h whereas Pkc1 and Tor1 putative sites increased (Fig. 6A, upper panel). A single motif ([pT/pS]P) was enriched among all phosphorylated sites, according to our MoMo analysis (Fig. 6B), and no specific motifs were enriched along time.

The profile of putative kinases assigned to dephosphorylated sites was more complex. Forty-three different kinases were mapped to the mentioned 203 sites, of which thirty-one could affect two or more sites (Fig. 6A, lower panel, Supplementary Table S6). Again, Tor1 and Pkc1 were the most prominent enzymes, but in this case Cdc28, Cka1, Cka2 and Pho85 also contributed significatively with at least five putative sites detected for each kinase. Interestingly, the time-course profile showed differences among kinases. Pkc1 and Tor1 putative sites were already found dephosphorylated after 1 h, but their number almost peaked at 2 h, with little additional increase after 4 h. Putative Cka1 and Cka2 sites were dephosphorylated after 2 h, and their number further increased after 4 h. In contrast, Cdc28 sites were not detected after 1 h, only one site was found after 2 h, whereas up to 14 possible sites were dephosphorylated at 4 h. To better map these dynamic changes, we used MoMo to visualize the newly appearing phosphosites (that is, removing from the list submitted to MoMo any site detected at a previous time-point). As can be observed in Fig. 6C, a single motif (RxxpS) emerged at 1 h, that likely responds to the early dephosphorylation of sites for basophilic kinases (such as Tpk1-3, Vhs1, Ypk1, Kin2, etc.). This motif was slightly altered at 2 h to RxxSxxpS, and to Sxx[pS/pT], likely reflecting the emergence of Cka1 and Cka2 enzymes. However, the major change can be observed after 4 h of Ppz1 induction (Fig. 6C). In this case, two major motifs emerged: [pS/pT]PxK, and a simpler one, [pS/pT]P, both clearly associated to Pro-directed kinases. This likely reflects the appearance of sites for new kinases such as Kss1 and Fus3, the increase (twofold) in Pho85 sites and, mainly, the dramatic increase in dephosphorylated putative Cdc28 sites. It should be noted that the highest enrichment (~ 38-fold) corresponds to the [pS/pT]PxK motif, which was defined earlier as a “strong” Cdc28 consensus site^38^.

### Rps6 is dephosphorylated upon overexpression of Ppz1

Recent work from our laboratory has pointed out that overexpression of Ppz1 causes depletion of polysomes and results in phosphorylation of eIF2α at Ser51^26^. Both effects are consistent with a halt in translation initiation. Our phosphoproteomic data indicated that, on top of several translation-related proteins (see above), a time-dependent dephosphorylation of Rps6A can be observed (Supplementary Fig. S2). Rps6A is a ribosomal protein whose phosphorylation is rapidly induced by nutrients in a TORC1-dependent manner^39^. To confirm this change, extracts from cells overexpressing Ppz1 were prepared and subjected to immunoblot to identify changes in the phosphorylation state of the relevant residues Ser232, 233. As observed in Supplementary Fig. S2, phosphorylation of Rps6A decreases as soon as detectable levels of Ppz1 appears. In fact, phosphorylation of Rps6A becomes unnoticeable after 2 h of Ppz1 overexpression, closely following the pattern derived from the phosphoproteomic analysis.

### Overexpression of Ppz1 triggers rapid Mig1 and Snf1 dephosphorylation

**Figure 7:**
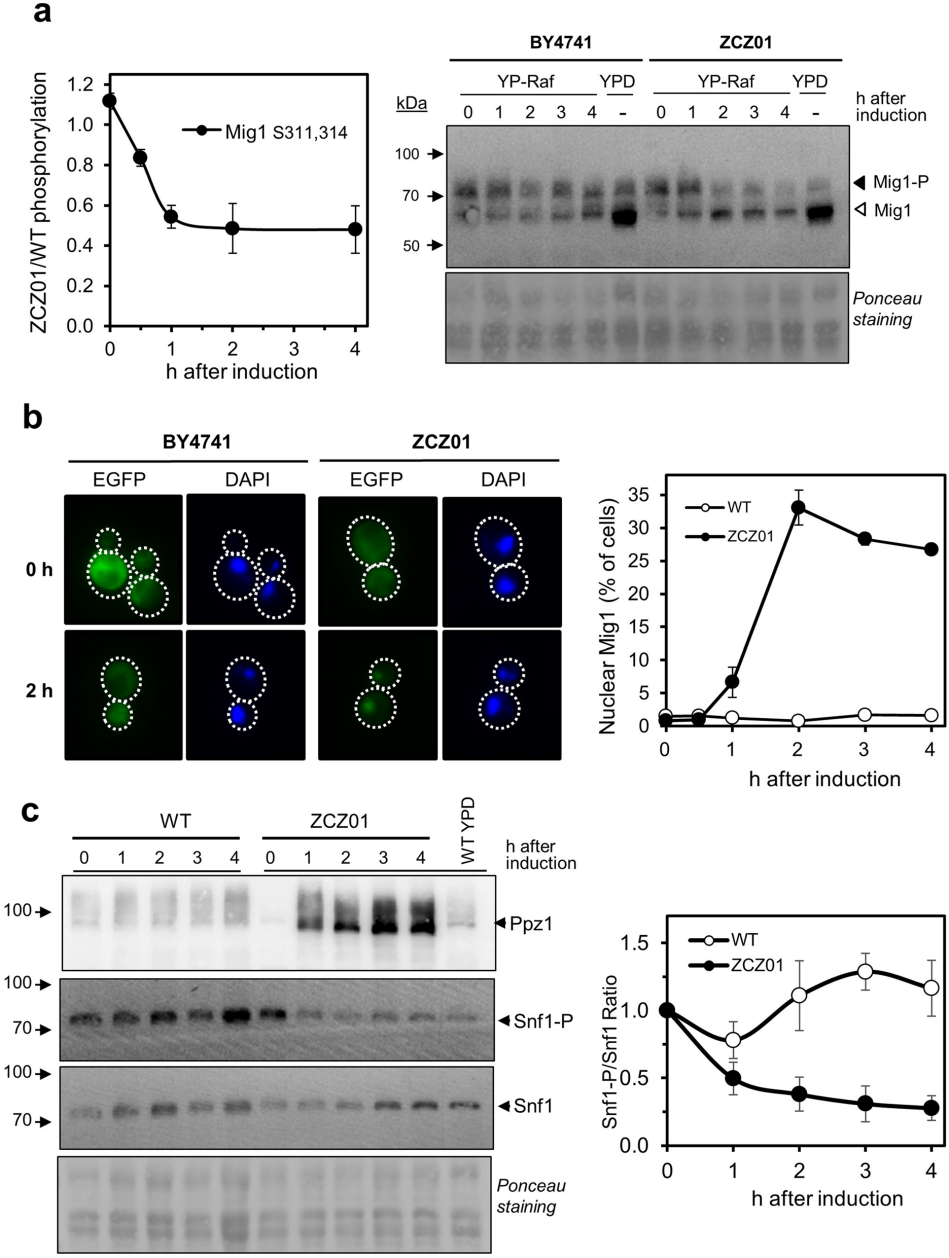
Dephosphorylation of Mig1 and Snf1 upon overexpression of Ppz1. (**a**) Left panel. Changes in phosphorylation of Mig1 at residues Ser311, 314 derived from the phosphoproteomic experiments. Data represent the mean ± SEM from 4 experiments. Right panel. Cells expressing a HA-tagged version of Mig1 were exposed to Ppz1 inducing conditions and the mobility of Mig1 monitored by SDS-PAGE (10% polyacrylamide) of protein extracts (40 µg of proteins) followed by immunoblot with anti-HA antibodies. Ponceau staining of the membrane is shown for comparison of loading and transfer efficiency. (**b**) Wild type (BY4741) and ZCZ01 cells were transformed with an episomal plasmid bearing a GFP-tagged version of Mig1. Cultures were treated for Ppz1 induction as described in Material and Methods. Cells were collected at different times, stained with DAPI to reveal the position of the nucleus, and observed in a fluorescence microscope. The graph on the right shows the abundance of cells (as %) in which Mig1 was retained in the nucleus. Data are the mean ± SEM (n = 3) from an average of 167 to 426 cells counted per experiment. (**c**) Changes in Snf1 phosphorylation induced by Ppz1 overexpression. Left panel. BY4741 (WT) and ZCZ01 cells were treated as in panel A and protein extracts electrophoresed, transferred to membranes and probed with anti-Ppz1, anti-AMPK-P^T172^ (Snf1-P), or anti-polyHis (Snf1) antibodies. Right panel. Quantification of the P-Snf1/Snf1 ratio by integration of the signals from three independent experiments. The result for the wild type strain at time = 0 is defined as the unit and the mean ± SEM is represented.

The rapid dephosphorylation of the transcriptional repressor Mig1 at Ser311,314 (Fig. 7A, left panel) attracted our attention, because these are physiological target residues for the Snf1 kinase. We first aimed to confirm by biochemical means the observed changes. Therefore, we transformed wild type BY4741 and ZCZ01 cells with a plasmid carrying a HA-tagged version of Mig1 and monitored by immunoblot changes induced in the electrophoretic mobility of the protein, known to be associated to changes in phosphorylation. As shown in Fig. 7A (right panel), one h after Ppz1 induction a fraction of Mig1 molecules migrates faster (dephosphorylated) and from 2 h onwards most of the signal is indistinguishable from that of cells growing on glucose (YPD lane), in which Mig1 is known to be dephosphorylated, thus confirming the phosphoproteomic data. Because dephosphorylation of Mig1 is usually associated to a nuclear localization of the transcription factor we investigated, by mean of a Mig1 version fused to GFP, possible changes in the intracellular localization of the repressor. As shown in Fig. 7B, Mig1 is largely cytosolic in wild type or ZCZ01 cells not exposed to galactose. However, from 2 h of addition of galactose onwards, while Mig1 was not affected in wild type cells, in around 30–35% of ZCZ01 cells the repressor localized to the nucleus. A similar behavior was observed in strain MLM04, where *PPZ1* is expressed from the *tetO*_7_ promoter in a galactose-free medium. In this case, shifting of cells to low glucose resulted in drastic reduction of nuclear Mig1 in wild type cells, while in Ppz1-overexpressing cells Mig1 remained in the nucleus (Supplementary Fig. S3).

Phosphorylation of Mig1 is usually linked to activation of the Snf1 kinase. Therefore, we aimed to investigate the phosphorylation status of this enzyme. As shown in Fig. 7C, Snf1 becomes dephosphorylated one h after induction of Ppz1, that is, when the amount of the phosphatase is still far from its highest levels. Reg1 is a regulatory subunit of the Glc7 protein phosphatase and is phosphorylated by Snf1 in response to low glucose. Our phosphoproteomic survey revealed multiple phosphorylation sites in Reg1, but only Ser346 and Ser349 were consistently dephosphorylated upon overexpression of Ppz1 (Supplementary Table S4 and Supplementary Fig. S4). Previous work in our laboratory showed that Ppz1 could dephosphorylate in vitro Reg1 at sites present within the first 443 residues^40^. Therefore, to confirm our phosphoproteomic data, we transformed wild type and ZCZ01 cells with a plasmid carrying a HA-tagged Reg1^1–443^ version and followed the change in electrophoretic mobility caused by dephosphorylation. As shown in Supplementary Fig. S4, overexpression of the phosphatase causes a shift of the Reg1^1–443^ protein to faster migrating species, already visible after 2 h. After 4 h in galactose, the mobility pattern of the Reg1^1–443^ protein was indistinguishable from that of cells growing on glucose, indicating substantial dephosphorylation.

### Changes in Hog1 and Sko1 phosphorylation could explain some effects derived from Ppz1 overexpression

**Figure 8:**
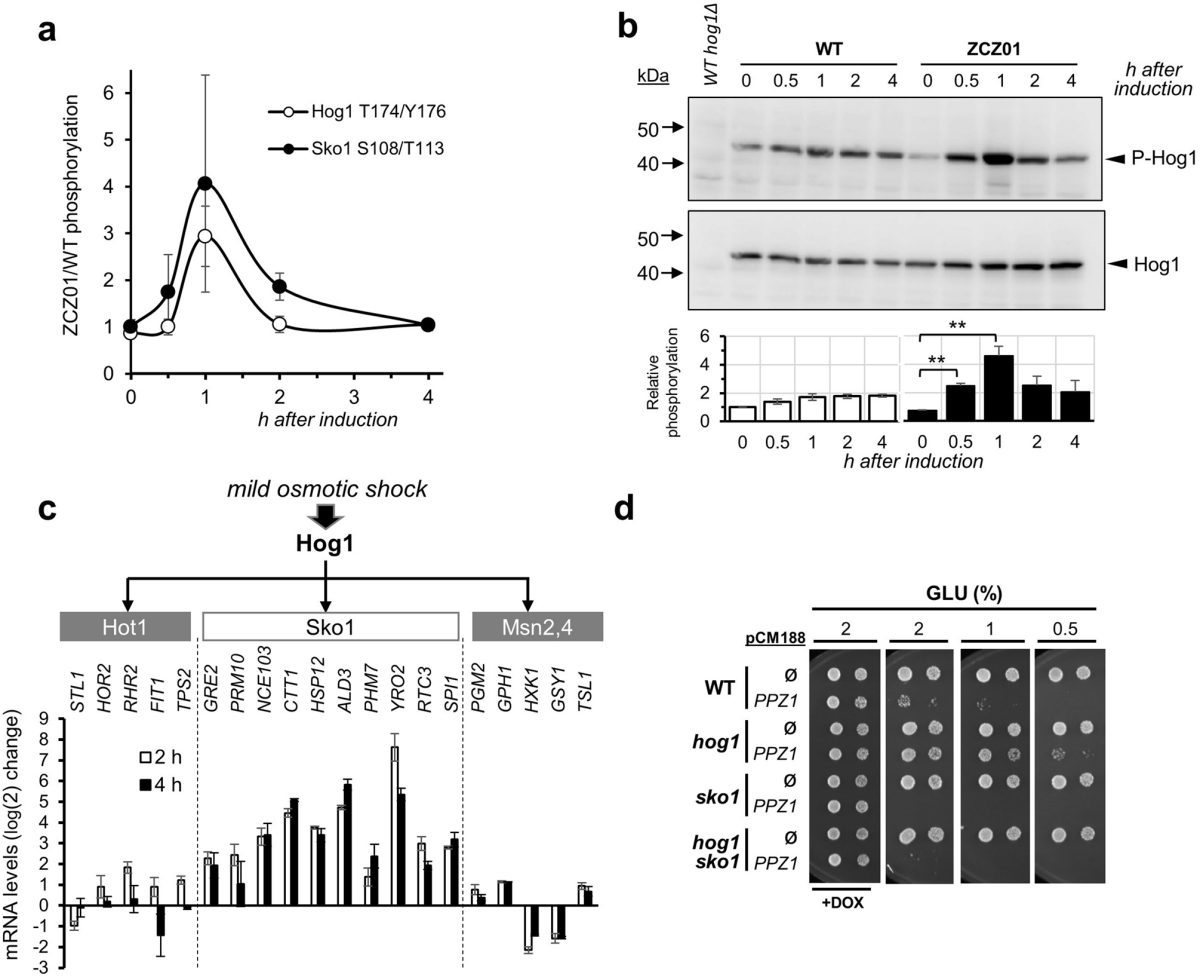
Hog1 and Sko1 mediate part of the signals elicited by overexpression of Ppz1. (**a**) Changes in phosphorylation of Hog1 (Thr174/Tyr176) and Sko1 (Ser108/Thr113) derived from the phosphoproteomic experiments. Data represent the mean ± SEM from two (Hog1) and four (Sko1) datasets. (**b**) Upper panel. BY4741 (WT) and ZCZ01 cultures in YP-Raff received 2% galactose to promote Ppz1 induction. Protein extracts (40 µg of proteins) were subjected to SDS-PAGE (10% polyacrylamide), transferred to membranes and immunoblotted with anti P-Hog1 (P-Thr174/Tyr176) antibodies. Upon detection, membranes were stripped and probed with anti Hog1 antibodies to detect the amount of total Hog1. Lower panel. Signals corresponding to P-Hog1 and Hog1 were integrated and the P-Hog1/Hog1 ratio calculated for each time point. The values from three independent experiments were combined and the result for the WT strain at time = 0 was taken as the unit. Data is expressed as mean ± SEM (n = 3). **p < 0.01 calculated by the Student’s t-test. (**c**) Twenty representative genes proposed to be induced by Hog1 activation (exposure to 0.375 M KCl) mainly due to involvement of the Hot1, Sko1 and Mns2/4 downstream components were selected based on data from Supplementary Table S2^42^ and the corresponding GEO entry (accession number GSE1227). The change in expression for these genes after 2 and 4 h upon induction of Ppz1 is plotted as the mean ± SEM (n = 3). (**d**) The indicated strains in the BY4741 background (WT) were transformed with plasmid pCM188-*PPZ1*. Overnight cultures were grown in SC medium lacking uracil with 100 µg/ml of doxycycline, washed twice with the same medium lacking doxycycline, resuspended in this medium and grown for five hours before spotting in plates with (+ DOX) or without doxycycline and containing the indicated amounts of glucose as carbon source. Pictures were taken after 3 days.

Examination of phosphoproteomic data revealed an increase of 2.9-fold in Hog1 phosphorylation at Thr174 and Tyr176 after one h of Ppz1 expression (Fig. 8A), although it was only detected in two experiments with a *p*-value of 0.08 (thus slightly above the 0.05 threshold). However, because phosphorylation of these sites is associated to activation of the Hog1 kinase, we sought to validate this change by immunoblot experiments using specific anti P-Thr174 and Tyr176 antibodies. As shown in Fig. 8B, phosphorylation of Hog1 increases upon Ppz1 overexpression and the quantitative change observed by immunoblot is reasonably coherent with that detected by mass spectrometry. The change is already evident at 30 min, peaks after one h, and phosphorylation levels remain somewhat higher after 4 h of induction compared to the pre-induction status.

Our data also showed increased phosphorylation of the transcription factor Sko1 at Ser108 and Thr113, which are sites known to be directly phosphorylated by Hog1 upon the activation of the kinase^41^. It is known that activation of Hog1 by mild osmotic stress results in profuse transcriptional changes that are in part mediated by Sko1^42^. Therefore, we sought to trace such effect in our transcriptomic data by comparing the transcriptional impact of Ppz1 overexpression with that of Hog1 activation, and considering the components downstream the kinase (Sko1, Hot, and Msn2/4) mediating this effect. As shown in Fig. 8C, Hog1-induced genes that are mediated by Hot1 or Msn2/4 according to Capaldi et al.^42^ were rarely induced (and sometimes even repressed) by Ppz1 overexpression. In contrast, the expression of those genes proposed to be under control of Sko1 clearly increased upon Ppz1 induction almost without exception. This suggested that the phosphorylation of the tandem Hog1-Sko1 might transmit, at least in part, signals elicited by overexpression of the phosphatase. To test such possibility, we transformed cells lacking *HOG1*, *SKO1* or both genes with the *tetO*_2_-based centromeric plasmid pCM188-*PPZ1*, which allows mild overexpression of the phosphatase in the absence of doxycycline. As it can be observed in Fig. 8D, and was recently reported^26^, expression of Ppz1 from pCM188 leads to a growth defect that is aggravated when glucose is less abundant. Under these conditions, deletion of *HOG1* clearly improved growth, while deletion of *SKO1* aggravated the growth defect. The double *sko1 hog1* mutant strains behaved as a *sko1* mutant, suggesting that Sko1 is acting downstream Hog1. Taking together, these data suggest that Hog1 contributes to the signaling that leads to the characteristic growth defect caused by Ppz1 overexpression.

## Discussion

Transcriptomic profiling of Ppz1-overexpressing cells showed a strong incidence in cellular mRNA levels, with over 1,300 genes (about 20% of the genome) exhibiting significative changes. This figure is even higher than that previously observed for genes responding to a large variety of stresses, the so called ESR (environmental stress response) or CER (Common Environmental Response)^30,43^, and suggests that overexpression of Ppz1 has a wide impact in many aspects of the yeast cell biology. These changes occur within a time span of 4 h after the shift to galactose of ZCZ01 cells. Our recent work using the same ZCZ01 strain model, in which DNA content and budding index was monitored, indicated a substantial impact on cell cycle two h after the shift to galactose and a full arrest with 1C DNA content after 4 h^26^, which is consistent with the massive gene expression remodeling observed. Previous work had revealed that overexpression of Ppz1 results in severe blockage of proliferation, with a halt at the G_1_ to S phase transition of the cell cycle and reduced expression of the G_1_ phase cyclins *CLN2* and *CLB5*^44^. Our transcriptomic data confirms these results and shows also repression of *CLN1* and *CLB1*. We observe in the short term (30 min) a modest but widespread induction of genes involved in ribosome biogenesis (> 50 genes). This period corresponds to the transition from virtually undetectable Ppz1 to levels that are barely above of those found in a wild type strain (see Fig. 1A and reference^26^), suggesting that this increase is congruent with the promotion of growth in exponentially growing cells (because control cells also receive the same amount of galactose, it cannot be attributed to the addition of a new carbon source). However, these genes are no longer induced at 2 h and, instead, overexpression of many genes responsive to oxidative stress occurs. Our data supports the notion that ROS levels increase in Ppz1-overexpressing cells (Fig. 2B) and our observation that this is accompanied by the formation of Rad52 foci, a common response to DNA-damaging conditions, is in keeping with the well-established notion that oxidative stress causes DNA damage^45^. It is worth noting that previous work reported that Ppz phosphatases are required for normal tolerance to oxidative stress not only in *S. cerevisiae*, but also in other fungi^46^.

**Figure 9:**
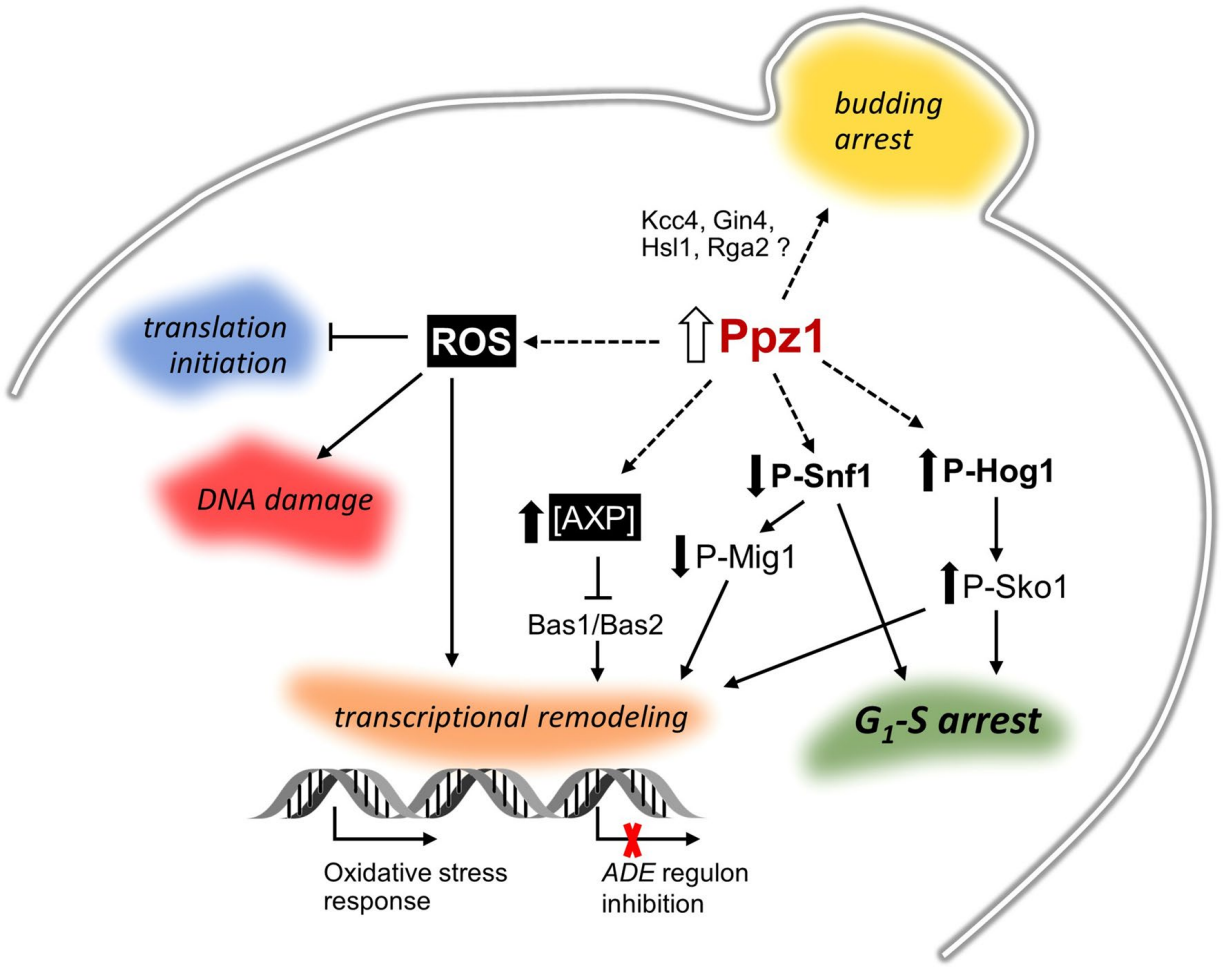
Cartoon depicting major effects of Ppz1 overexpression discussed in this work. Discontinuous lines denote uncertainty about the precise mechanism leading to the indicated effect.

An important effect of oxidative stress is the inhibition of translation at the initiation and post-initiation phases. This is interesting because we recently reported^26^ that overexpression of Ppz1 results in a halt in protein translation, with a reduction in polysome content. It has been described that the response to oxidative stress is mediated by oxidant-specific regulation of translation initiation, in a way that inhibition of translation initiation in response to hydroperoxides is entirely dependent on phosphorylation of eIF2α by the Gcn2 kinase, while in response to cadmium and diamide also requires the participation of the eIF4E binding protein Eap1^47^. We have previously shown that overexpression of Ppz1 results in hyperphosphorylation of eIF2α at the conserved Ser51 residue and that deletion of *GCN2* (but not that of *EAP1*) attenuates somewhat the growth defect of cells overexpressing Ppz1^26^. This suggests that the oxidative stress suffered by cells that overexpress Ppz1 might contribute to the observed halt in protein translation initiation (Fig. 9). In addition, such intracellular stress would be reminiscent to that generated by exposure of the cells to hydroperoxides, such as hydrogen peroxide. This compound can be formed as by-product of aerobic respiration. It is tempting to speculate that the observed widespread decrease in the expression of genes encoding mitochondrial ribosomal proteins, as well as regulatory components of respiration (Fig. 1B), might be linked to the observed generation of ROS. In any case, the reported impact on translation likely justifies the relatively minor overall change in protein levels in spite of the robust transcriptional reaction to increased levels of Ppz1.

Mapping of transcriptomic data onto the YeastPathways database revealed diverse metabolic pathways with a high Pathway Perturbation Score (PPS, (Supplementary Fig. S1)). The highest score corresponded to the pathway leading to synthesis of β-alanine from spermine^48,49^, based on the induction of *FMS1*, *ALD2* and *ALD3* genes. It is worth noting that, in spite of the limited changes in the proteome, in addition to Ald3, a small but significant increase in Ald2 could be detected (1.56-fold, *p*-value 0.001). β-alanine is an intermediate required for the de novo biosynthesis of pantothenic acid and hence for CoA biosynthesis. Because the regulatory subunits of Ppz1 are also involved in CoA biosynthesis^20^ it is plausible that the observed transcriptional effect could be related to a possible impact of the overexpression of Ppz1 on the biosynthesis of this coenzyme. It must be stressed, however, that this hypothetic effect cannot explain the growth defect of Ppz1-overexpressing cells^26^. Interestingly, genes *TPO2* and *TPO3*, encoding specific spermine transporters, were also induced and both proteins experienced strong changes in their phosphorylation state (albeit, as far as we know, no evidence for phospho-regulation of these transporters has been reported).

Inosine monophosphate (IMP) biosynthesis, required for the de novo biosynthesis of purine nucleotides (the *ADE* pathway), was another pathway highly perturbed by overexpression of Ppz1 (Fig. 1B,C; Supplementary Fig. S1), since virtually all genes of the pathway, with the exception of *ADE16*, were down-regulated (Fig. 3A). This exception was interesting because *ADE16* is the only gene in the pathway that is not under the control of the Bas1-Bas2 transcription factor complex^34,50^. It has been shown that oxidative stress causes down-regulation of the *ADE* pathway by blocking the necessary Bas1-Bas2 interaction^34^. Therefore, we conjectured that the oxidative stress observed in Ppz1-overexpressing cells could be responsible for down-regulation of the *ADE* pathway, leading to depletion of adenine nucleotides. Because asynchronous growing yeasts cells largely relay in de novo synthesis of dNTPs to enter in S-phase^51^, we hypothesized that this effect might explain the cell cycle blockage observed in Ppz1-overexpressing cells. However, in contrast to our hypothesis, the levels of dNTPs were actually higher in cells overexpressing Ppz1 than in control cells (Fig. 3C). In fact, when the levels of ribonucleotides were investigated, we found a clear-cut increase in ATP and GTP levels (Fig. 3B), as well as in the adenylate pools (AXP) and in the adenylic energy charge (AEC). Consistent with this scenario^52^, the levels of NAD^+^ also increased. Therefore, downregulation of the *ADE* pathway could be explained by a negative feed-back caused by the accumulation of its final products (Fig. 9). In favor of this hypothesis is the markedly lower level of AICAR monophosphate (ZMP) in Ppz1-overexpresing cells. This important intermediate has been shown to be a major activator of the expression of the Bas1-Bas2 dependent genes in the pathway^53^.

Whereas changes in AEC have been found associated to many physiological or stressful conditions, changes in the amount of AXP are far uncommon. A reduction of ~ 30% in the total adenine nucleotide pool was reported^54^ in cells exposed to toxic alkali cations (Li^+^ and Na^+^, but not to K^+^), but the origin was not deciphered. In contrast, an increase in AXP was reported by Szijgyarto et al.^55^ as a result of depletion of inositol pyrophosphates caused by deletion of *ARG82* and/or *KCS1*. However, we do not observe downregulation of these genes, nor detect in our transcriptomic data the reported changes in the expression pattern for several glycolytic genes resulting from their inactivation (in particular, *ADH2* activity decreases fivefold in a *kcs1*Δ mutant, whereas it is potently induced in Ppz1-overexpressing cells). At this moment we cannot provide an explanation for this accumulation of AXP molecules, but it is evident that the failure to proliferate of cells with high levels of Ppz1 is not due to lack of energy or building blocks for DNA synthesis.

We show here that Ppz1 overexpression alters numerous (> 150) phosphoproteins, most of them by dephosphorylation of one or more phosphosites, indicating a wide impact on the cell biology. This is in contrast with the almost null effect on the phosphoproteome observed upon deletion of *PPZ1* by Bodenmiller et al.^56^. It is worth noting that, in response to Ppz1 overexpression, diverse protein kinases are phosphorylated (Ksp1) or dephosphorylated (Kcc4, Gin4, Hsl1, Rck2, Tda1 and Npr1). Kcc4, Gin4, Hsl1 are involved in septin organization and checkpoint required for proper bud formation, a process that is blocked in Ppz1-overexpressing cells (Fig. 9). Interestingly, expression of all three kinases is also downregulated (Supplementary Table S1). It is worth noting that Rga2, a GTPase-activating protein also implicated in the control of septin organization, is dephosphorylated in Ppz1-overexpressing cells at several sites that are known targets for Pho85-Pcl complexes and Cdc28 kinase^57^.

Similarly, several protein phosphatases are affected, mostly at their regulatory subunits. Thus, Gip2 (Glc7), and Sap155 (Sit4) subunits are phosphorylated, while Reg1, Bni4, and Gac1 (all three Glc7 subunits), and Rts1 (Pph21/22) are dephosphorylated. This could likely amplify the effect of overexpression of Ppz1 on the phosphoproteome. Indeed, some of the observed changes (not only phosphorylation events) could be indirect effects. This might be the case for the dephosphorylation of Rps6A at Ser232, 233, which has been previously attributed to Glc7 associated to the Shp1 subunit^58^, although we cannot discard that those residues could be also direct targets for Ppz1.

The output of analysis of putative kinases able to phosphorylate sites affected by Ppz1 overexpression, particularly those that are dephosphorylated, was intriguing. Early dephosphorylated sites were enriched in Arg-directed residues, which are characteristic of PKA and other metabolic kinases, while at later time Pro-directed residues, typical for Cdc28 kinase were dephosphorylated. It must be noted that these motifs are almost identical to those identified by Zhang et al.^59^ as being phosphorylated in cells resuming cell cycle after G_1_ arrest. This similarity remarks the massive effect of Ppz1 overexpression in modifying cell cycle-regulated phosphoproteins.

Since it is plausible that more direct Ppz1 effects would result in early cellular changes, we focused in specific variations occurring shortly after Ppz1 overproduction (60 min). At this time Snf1 is dephosphorylated at T210, likely inhibiting its activity, and this possibly explains the dephosphorylation of Mig1 at Ser311 and its reluctance to abandon the nucleus^60,61^. We also observe the dephosphorylation of Hxk2 at Ser15. Hxk2 is the major glucose kinase in high glucose conditions, but it also acts as a regulator of gene transcription in the nucleus. When there is plenty of glucose, Hxk2 interacts with Mig1 at Ser311, avoiding Mig1 phosphorylation by Snf1 and the exclusion of the repressor from the nucleus. Remarkably, phosphorylation of Hxk2 Ser15 by Snf1 prevents nuclear localization of Hxk2 and hence its interaction with transcription factors^62^. Thus, dephosphorylated Hxk2 tends to remain in the nucleus and to collaborate with Mig1 in repressing transcription. This model agrees with our observation that the mRNA levels of diverse targets affected by Hxk2, such as *HXK1*, *GLK1* or *SUC2* (see^63^ and references therein) decrease with Ppz1 overexpression (Supplementary Table S1).

Reversion of the effects of Snf1 on Mig1 and Hxk2 are attributed to the Glc7 protein phosphatase in association with its targeting subunit Reg1^62,64^. Reg1 is phosphorylated in vivo at more than 50 sites, many of them also identified in our analysis. Interestingly, overexpression of Ppz1 specifically resulted in dephosphorylation of only two sites, Ser346 and Ser349, that are included within the Reg1^1–443^ polypeptide. We show here that this protein undergoes a shift to higher mobility (known to be dephosphorylated) species when Ppz1 is overexpressed, suggesting that this change might be caused by dephosphorylation of the mentioned residues. A similar shift was observed when this fragment was used for in vitro dephosphorylation assays using recombinant Ppz1^40^. Therefore, although dephosphorylation of Reg1 has been usually attributed to Glc7^65^, our data suggest that Reg1 might also be an in vivo substrate for Ppz1. At present, we don’t know if the dephosphorylation of Ser346 and Ser349 has any effect on Reg1 function. It is worth noting that retention of Mig1 in the nucleus cannot explain the growth defect of Ppz1-overexpressing cells, since we have observed that deletion of *MIG1* does not solve the problem, and that of both *MIG1* and *MIG2* even aggravates it (see Supplementary Fig. S3B). It must be stressed, however, that a role for Snf1 in cell cycle regulation has emerged in the last few years^66,67^. Thus, it was found that inhibition of Snf1 activity results in an increased fraction of cells in G_1_ phase, in a way that was dependent on the nutritional composition of the media, and in reduced expression of G_1_-specific genes (including *CLB5*). Furthermore, the non-phosphorylatable Snf1-T210A mutant shows a slow growth phenotype and a delayed G_1_/S transition, and these phenotypes can be fully rescued by expression of the phosphomimetic (T210E) form of Snf1. Very recently, it has been reported that Snf1 becomes dephosphorylated at T210 during G_1_ to S phase transition but becomes phosphorylated again at the onset of DNA synthesis^59,68^. Therefore, it is plausible to assume that continuous dephosphorylation of Snf1 at Thr210 forced by overexpression of Ppz1 might contribute to the blockage at the G_1_-S transition in these cells (Fig. 9).

We have shown that overexpression of Ppz1 results in increased Hog1 phosphorylation at the activating Thr174 and Tyr176 residues, and that deletion of this kinase improves growth in Ppz1-overexpressing cells (Fig. 8D). This suggests that activation of Hog1 mediates in part the effect on cell growth caused by high levels of the phosphatase. It has been shown that Hog1 activation induces a delay in the G_1_-S transition, and that this occurs by at least two mechanisms: phosphorylation of the CDK inhibitor Sic1 at Thr173^69^ or inhibiting G_1_ cyclin transcription by targeting Whi5 (a repressor of G_1_ transcription) and Msa1/2 transcription factors^70^. We have not identified Sic1 phosphopeptides containing phosphorylated Thr173, nor detected increased phosphorylation in Ser88 or Thr142, two of the three presumed Hog1 targets in Whi5^70^. In contrast, we observe a clear dephosphorylation of Whi5 at Ser154, Ser156, and Ser161, three out of the four residues relevant for inactivation of this repressor that are targets for Cdc28^71,72^. Therefore, it must be assumed that the impact of Hog1 in Ppz1-overexpressing cells might follow alternative paths. In any case, our data shows a functional hierarchy between Hog1 and Sko1 in cells with high levels of Ppz1, since deletion of *SKO1* results in lack of growth even if Hog1 is removed (Fig. 8D). A possible interpretation is that the unphosphorylated form of Sko1 would have a significant positive effect on cell growth. Activation of Hog1 by high Ppz1 levels would displace part of the Sko1 population to a phosphorylated form that would no longer exert the presumed positive effect. In fact, this might even have negative consequences, as it has been shown that phosphorylation by Hog1 converts Sko1 from a repressor into an activator^73^. In these circumstances, deletion of *HOG1* would impede Sko1 phosphorylation, thus improving growth. Lack of Sko1 would eliminate its positive impact on growth in Ppz1-overexpressing cells, and this would happen irrespectively of the presence of Hog1 (Fig. 9). Niu et al. reported that strong overexpression of *SKO1* strongly inhibited cell growth and arrested cells at the G_1_ phase^74^. Although these results seem to be in contradiction with those obtained here, it must be noted that the growth arrest effect described by these authors was associated to a likely spurious activation of the pheromone response pathway, which do not appear to be regulated by Sko1 under normal culture conditions^74^.

In conclusion, our work shows that overexpression of the Ppz1 phosphatase causes a myriad of cellular alterations. We recently demonstrated that deletion of *GCN2* or overexpression of genes encoding ribosomal proteins partially counteracts the growth defect caused by high levels of Ppz1^26^. We show here a similar effect by deletion of the *HOG1* kinase, as well as an extensive impact on gene expression and protein phosphorylation. Therefore, the emerging scenario suggest that the toxicity of Ppz1 is not caused by the deregulation of a single specific target, but by the accumulation of alterations in multiple cellular processes.

## Materials and methods

### Growth of *Escherichia coli* and yeast strains

Unless otherwise stated, yeast cells were incubated at 28 °C in YP medium (1% yeast extract, 2% peptone) or in synthetic medium (SC) lacking the appropriate selection requirements^75^. Carbon sources such as glucose (Glu, in YPD), raffinose (Raff, YP-Raff) or galactose (Gal, YP-Gal), were added as indicated at 2% unless otherwise stated. Plates contained 2% agar. Yeast strains used in this work and the construction methods are described in S1 File. Transformed yeast cells containing *PPZ1* under the control of doxycycline-repressible promoters were plated always in medium containing doxycycline (100 µg/ml). *E. coli* DH5α cells were used as plasmid DNA host and were grown at 37 °C in LB medium supplemented with 50 μg/ml ampicillin, when required. Transformations of *S. cerevisiae* and *E. coli*, and standard recombinant DNA techniques were performed as previously described^76^.

### Plasmids

Plasmids pCM188-*PPZ1* and pCM189-*PPZ1* are centromeric *tet-off* vectors that express *PPZ1* from a *tetO*_2_- or *tetO*_7_-driven promoter^26^. Plasmid pCM190-*PPZ1* is an episomal, *tetO*_7_-driven vector^26^. Plasmid B273 is a centromeric vector (*URA3* marker) expressing, from the *BAS1* promoter, a chimera fusing the 1–705 N-terminal residues of Bas1 to the entire Bas2 protein^77^. Expression of this chimera leads to constitutive expression of most genes of the *ADE* pathway^34,77^. Plasmid pWJ1344 is a pRS415-based vector expressing Rad52-YFP from its own promoter^78^. Expression of Mig1 fused to GFP was accomplished by transforming cells with plasmid YEp195-GFP-Mig1. pHA-Mig1 is a pRS426 derivative carrying a HA-tagged version of Mig1^79^. pREG1-HA^(1–443)^ derives from the pWS93 multicopy plasmid (*URA3* marker) and carries a 3xHA-fusion with the N-terminal region (residues 1–443) of Reg1^65^. Plasmid pGAP1-LacZ is described in^80^, plasmid pGRE2-LacZ can be found in^81^, and plasmid pSIT1-LacZ was reported in^7^. For construction of pNCE103-LacZ, the *NCE103* promoter was amplified from genomic DNA by PCR with oligonucleotides NCE103_prom_5′ and NCE103_prom_3′ and cloned into the EcoRI-HindIII sites of vector YEp357.

### RNA purification and RNA-Seq analysis

For the analysis of the transcriptional response to Ppz1 overexpression induced by galactose, wild type BY4741 and ZCZ01 (*GAL1:PPZ1*) strains were grown overnight on YPD medium, and then cells were transferred to medium with raffinose as carbon source at starting OD_600_ of 0.2. When the OD_600_ of the cultures reached around 0.6, galactose was added to a final concentration of 2%. Samples (10 ml) were taken after 30, 120 and 240 min by quick filtration through GN-6 Metricel filters (Pall Corp.), immediately frozen and total RNA was extracted and characterized as in^82^.

Library preparation, RNA sequencing and data processing was done essentially as in^83^ although in this case single end, 75 nt/read were obtained. Three replicates were sequenced, yielding a total number of 3.7–8.3 million reads per condition, of which 97.2–98.6% were mapped to the *S. cerevisiae* genome. Raw data was filtered to remove sequences with low number of reads. The Pearson’s correlation coefficient between replicates ranged from 0.874 to 0.998 (averages values of 0.970, 0.958 and 0.917 for data at 0.5, 2 and 4 h, respectively). Genes were considered induced or repressed upon Ppz1 overexpression when changes for a given time-point were at least twofold (log(2) ≥ 1 or log(2) ≤ − 1) with a *p*-value < 0.05 (paired t-test). Data can be retrieved from the Gene Expression Omnibus (GEO) repository under study GSE138750.

Transcriptomic data was mapped onto metabolic pathways using the Omics Viewer at the SGD YeastPathways service (https://pathway.yeastgenome.org), which was built using the Pathway Tools software^29^. Pathway Perturbation Score (PPS) measures the overall extent to which a pathway is up- or down-regulated, by combining the activation levels of all reactions in the pathway. Differential PPS (DPPS) computes the maximum extent to which a pathway is perturbed between time-points. See main text for details. Gene Ontology analyses were done at the YeastMine WEB site with default settings^84^.

### LacZ-reporter assays

For *LacZ*-reporter assays, wild type BY4741 and ZCZ01 cells were transformed with the relevant plasmids and cultures were prepared as for RNA-Seq, except that samples (1.5 ml) were taken after 2 and 4 h of galactose addition, collected by centrifugation and stored at − 80 °C. The β-galactosidase activity was determined in permeabilized cells as described in^85^. Three individual clones were tested.

### Metabolomic determinations

BY4741 and ZCZ01 cells were exponentially grown in raffinose (until OD_600_ = 0.6) and then made 2% galactose at time 0. Twenty ml culture (around 2 × 10^7^ cells/ml) were taken at the indicated times and processed for monitoring levels of nucleotide derivatives using ion-exchange chromatography with UV detection as in^52^. Four to six independent extractions were done, and metabolic extracts were normalized to cell number and cell median volume using a Multisizer IV Beckman Coulter Counter. Metabolite concentrations were then determined by using standard curves with pure compounds. AXP corresponds to the sum of adenylic nucleotide contents: [AXP] = [ATP] + [ADP] + [AMP]. Adenylic energy charge was defined as AEC = ([ATP] + ½ [ADP])/[AXP])^86^.

Determination of dNTP levels was performed as follows. Cells were grown as indicated above and five OD_600_ (≈ 10^8^ cells) were collected by filtration on GN-6 Metricel (47 mm, 0.45 μm, Gelman Sciences) membranes. Samples were then processed essentially as in^87^ and dNTP content determined by a polymerase‐based method as described in^88^.

### Microscopy and flow cytometry-based techniques

To evaluate ROS formation cells were grown on YP with 2% raffinose up to OD_600_ = 0.6. Then, galactose was added to reach a concentration of 2% and, at the same time, dihydrorhodamine 123 (Sigma) was added at 2.5 μg/ml. Samples (1 ml) were taken after 2, 4 and 6 h and fixed with formaldehyde (2%) for 5 min. Samples were subjected to microscopic observation using a FITC filter. Aliquots (10 μl) were added to 1 ml of PBS solution and analyzed in a FACSCanto (Becton & Dickinson Co.) flow cytometer.

For determination of Rad52 foci, cells were transformed with plasmid pWJ1344, expressing Rad52-YFP from its own promoter^78^. Transformants were grown on synthetic medium lacking leucine with 2% raffinose until OD_600_ = 0.6 and then galactose was added up to 2%. Aliquots were taken at different times and fixed as above prior microscopic observation in a Nikon Eclipse TE2000-E microscope with a custom filter (excitation wavelength at 500 nm and emission at 535 nm).

For subcellular localization studies of Mig1, cells were grown as for ROS determination. Upon induction of Ppz1 expression, cells were collected at specific times, fixed with 2% formaldehyde and washed twice with PBS. To perform nuclear staining, cells were incubated with DAPI at 0.3 µg/ml for 30 min (ZCZ01 cells induced for 2 or more hours required a longer incubation time, 120 min). After the incubation, cells were washed twice with PBS and resuspended in mounting solution (0.1% *p*-phenylenediamine in PBS (w/v), 70% glycerol). Images were taken using a Nikon Eclipse TE2000-E microscope. Nuclear Mig1 localization was assigned by co-localization with DAPI stained material.

### Sample collection and preparation of protein extracts for immunoblot

Yeast crude extracts for immunodetection were prepared as follows. For monitoring Ppz1 levels in the ZCZ01 strain, or in BY4741 cells carrying the pYES2-*PPZ1* plasmid, cells were incubated at 28 °C in YP Raffinose (2%) until OD_600_ 0.6–0.8. Ten ml samples were collected before and at specified time-points after addition of galactose. Protein extracts were prepared essentially as described in^22^. Cell pellets were resuspended in 125 µl of Lysis Buffer A (50 mM Tris–HCl pH 7.5, 150 mM NaCl, 10% Glycerol, and EDTA-free Protease Inhibitor Cocktail (Roche)) supplemented with 0.1% Triton X-100, and 2 mM dithiothreitol (DTT). Cells were disrupted by vigorous shaking using a FastPrep cell breaker at setting 5.5 for 45 s (3 cycles) after addition of 125 µl of Zirconia 0.5 mm beads. Samples were centrifuged at 500×*g* for 10 min at 4 °C and protein concentration of the cleared supernatants quantified by the Bradford method (Sigma Chemical Co). To analyze the phosphorylation state of Rps6A, cells were collected and processed as above, except that Lysis Buffer B (50 mM Tris–HCl pH 7.5, 150 mM NaCl, 15% glycerol, 0.5% Tween-20, 1 × phosphatase inhibitor cocktail Set V (Millipore), 1 mM PMSF and 1 × EDTA-free protease inhibitor cocktail (Roche)) was used. Protein extracts to detect phosphorylated Snf1, total Snf1 protein, and the mobility shift of Reg1 were prepared by treating cultures (5 to 10 ml) with trichloroacetic acid (TCA) as described in^89^. Extracts for Mig1 were prepared as described for Sch9 in^90^, except that the NTCB cleavage step was omitted.

### SDS-PAGE and immunodetection

Unless otherwise stated, protein extracts were mixed with 4 × SDS-PAGE loading buffer to yield a final concentration of 1x, heated for 5 min at 95 °C, and resolved by SDS-PAGE at a concentration of 10% polyacrylamide. Proteins were transferred onto polyvinylidene difluoride (PVDF) membranes (Immobilon-P, Millipore).

Ppz1 was detected using polyclonal anti GST-Ppz1 antibodies^1^, at a 1:250 dilution. Phosphorylated Snf1 was monitored with anti-phospho-Thr172-AMPK (Cell Signaling Technology) at 1:1,000 dilution in TBST (Tris-buffered saline plus 0.1% Tween-20) supplemented with 5% BSA. To detect the phosphorylated Rps6A protein, an anti-Phospho-S6 Ribosomal Protein (Ser235/236) (Cell Signaling) antibody was used diluted 1:2000 in the solution described above. Immuno-identification of the phosphorylated form of Hog1 was accomplished with anti-phospho p38 (Thr180/Tyr182) antibodies (Cell Signaling). In these experiments, a secondary anti-rabbit IgG-horseradish peroxidase antibody (GE Healthcare) was used at 1:10,000–20,000 dilution.

Total Snf1 protein was identified with anti-polyHis (Merck, #H1029) antibodies, whereas HA-tagged Mig1 and Reg1 proteins were detected using anti-HA antibodies developed in mouse (Biolegend, #901502), in all cases at 1:1,000 dilution. Total Hog1 was detected with a monoclonal antibody raised in mouse (Santa Cruz Biotechnology). In these cases, secondary anti mouse IgG-horseradish peroxidase antibodies (GE Healthcare) were employed at a 1:10,000 dilution.

Immunoreactive proteins were revealed using the ECL Prime Western blotting detection kit (GE Healthcare) and an Imaging-System-Versadoc 4,000 MP (BioRad) apparatus. Membranes were stained with Ponceau Red to monitor sample loading and transfer efficiency.

### Phosphoproteomic analysis

Yeast extracts for phosphoproteomic assay were prepared as follows. Two hundred fifty ml of cells (BY4741 and ZCZ01 strains) were incubated from OD_600_ 0.2 until OD_600_ of 0.6–0.8 in YP Raffinose (2%). At this point, one sample (50 ml) for each strain was collected (T0) and galactose was added to the rest of the culture to a final concentration of 2%. Then 50 ml-aliquots were taken from each culture at 30, 60, 120 and 240 min. In each case, the cultures were mixed with TCA (final concentration 6%) and maintained on ice for at least 10 min. Cells were then pelleted at 1680×*g* for 5 min. The pellet was washed twice with 1 ml of cold acetone, dried at 37 °C, and finally frozen at − 80 °C. Protein extraction, phosphopeptide preparation and TMT labeling are described in Supplementary File S1.

The labeled samples were mixed so that the minimum ratio between MS2 reporter ion channels was > 80% in average for all peptides. Twenty μg of the mixed TMT sample was taken for protein normalization among samples. The sample was fractionated by high pH reversed-phase (HpHRP) into ten fractions, which were concatenated into five fractions for LC–MS analysis (fraction 1 with fraction 6, fraction 2 with fraction 7 etc.) to generate the proteomic set of data. The rest of the TMT mixed sample (800–900 μg) was enriched for phosphopeptides with TiO_2_ beads (GL Sciences, Tokyo, Japan) in experiment 1, or an IMAC column in experiments 2 to 4. The phosphopeptide mixture was similarly pre-fractionated with HpHRP columns into five final fractions.

LC–MS analysis was carried out using an Ultimate 3000 RSLCnano HPLC system connected to a Q-Exactive HF mass spectrometer (ThermoFisher Scientific). Samples were loaded on a PepMap 100 C18 (5 µm, 0.3 × 5 mm) Trap Cartridge (ThermoScientific) in loading solvent (2% acetonitrile, 98% H_2_O, 0.1% TFA) at a flow of 10 μl/min and separated in a fused-silica column (75 μm × 30 cm) home-packed^91^ with Reprosil Pur C18AQ 1.9 sorbent (Dr. Maisch). Samples were eluted with a 1 h linear gradient from 6 to 32% of solvent B (99% acetonitrile, 0.1% formic acid) in solvent A (99.9% H_2_O, 0.1% formic acid) at 0.27 μl/min flow at RT. Between each set of fractions the system was conditioned using a BSA tryptic digest (50 fmol) in a 15 min LC gradient. Data collection is described in Supplementary File S1.

Raw data was analyzed using the MaxQuant software (v1.6.3.3) and its integrated search engine Andromeda^92^ with the following parameters: Reporter ion MS2 (TMT11plex search), PSM FDR and protein FDR were set at 0.01; minimum peptide length was 7; minimum score for modified peptides was 40, and razor peptides (set to 1) were used for protein quantification. The search was executed against the *S. cerevisiae* “orf_coding.fasta” database (https://www.yeastgenome.org/). For protein abundance analysis, the ProteinGroups.txt MaxQuant file was used. For phosphoproteomic analysis the Phospho (STY)Sites.txt file was generated. To account for possible changes in the level of specific proteins, the relevant data from the Phospho (STY)Sites.txt file was normalized with the protein abundance data extracted from the ProteinGroups.txt file. Only phosphosites with localization probability ≥ 0.7 were selected and further processed with the Perseus software (v1.6.2.3)^93^. The PTM localization score is the probability for a given phosphosite in a phosphopeptide (a ≥ 0.7 threshold means that the added probability of all other potential phosphosites is 0.3), and corresponds to a false localization rate < 4%, according to Ferries et al.^35^.

To evaluate changes in protein abundance, data was loaded in Perseus, contaminants were removed and the Ppz1 overexpression/control ratios for each experiment were calculated. Categorical annotation and grouping of samples by experiments was done. Then, mean values and SEM were calculated and a 2-sample *t*-test (*p* < 0.05) was performed to identify consistent changes. A similar process was carried out for phosphopeptide analysis, with a *p* < 0.05 value set for significance.

### Motif discovery and identification of kinases

Motif enrichment was carried out with the MoMo software (https://meme-suite.org/tools/momo), on the MEME suite^94,95^ with the following settings: algorithm, motif-x; background peptides extracted from reference sequence (GenBank GCF_000146045.2_R64_protein.faa); motif width, 15; Combine motifs with different central residues with the same modification mass; *p*-value threshold, 0.0001.

The evaluation of the possible protein kinases targeting sites whose phosphorylation state was affected by overexpression of Ppz1 was done with NetworKIN 3.0 (https://networkin.info/index.shtml)^96^. The yeast Ensemble 74 data base was used, and min score was set to 2. When more than one kinase was assigned to a given site, the kinase with the highest score was selected.

## Supplementary information

Supplementary file1

Supplementary file2

Supplementary file3

Supplementary file4

Supplementary file5

Supplementary file6

Supplementary file7

## Data Availability

The datasets produced in this study are available in the following databases: RNA-Seq data can be retrieved from the Gene Expression Omnibus (GEO) repository under study GSE138750. Mass spectrometry proteomics data have been deposited to the ProteomeXchange Consortium via the PRIDE^97^ partner repository with the dataset identifier PXD018100.
