# Supplementary material for: Yeast Ppz1 protein phosphatase toxicity involves the alteration of multiple cellular targets
Source: Sci Rep. 2020 Sep 24;10:15613. doi: 10.1038/s41598-020-72391-y (PMC7519054; doi:10.1038/s41598-020-72391-y)
Supplement: Supplementary file 7 — Supplementary file7 [file 41598_2020_72391_MOESM7_ESM.pdf]

Supplementary File S1

Yeast Ppz1 protein phosphatase toxicity involves the alteration of multiple cellular targets

Diego Velázquez, Marcel Albacar, Chunyi Zhang, Carlos Calafí, María López-Malo, Javier Torres-Torronteras, Ramón Martí, Sergey I. Kovalchuk, Benoit Pinson, Ole N. Jensen, Bertrand Daignan-Fornier, Antonio Casamayor, Joaquín Ariño

Contents:

1. Yeast strains used in this work
2. Oligonucleotides used in this work
3. Proteomic and phosphoproteomic methods (additional)
4. Supplementary Figures S1-4
5.- Full-length gels and immunoblots presented in this work.

1. Yeast strains used in this work

The genotype of the strains used in this work is described in the following table.

Name	Features	Reference or source
BY4741	MAT **a** *his3*Δ*1 leu2*Δ *met15*Δ *ura3*Δ	[1]
ZCZ01	BY4741 $_{p}$*GAL1-10:PPZ1*	[2]
MLM04	BY4741 $_{p}$*tetO*$_7$*:PPZ1*	[2]
CCP018	BY4741 *hog1::natMX4*	This study
CCP020	BY4741 *sko1::kanMX4*	This study
CCP021	BY4741 *hog1::natMX4 sko1::kanMX4*	This study
SP038	BY4741 *mig1*::*kanM*X4	This study
SP043	BY4742 *mig2*::*kanM*X4	This study
SP010	BY4741 *mig1*::*kanM*X4 *mig2*::*kanM*X4	This study

Strain ZCZ01 and MLM04, which express *PPZ1* from the *GAL1* and a *tetO*$_7$ promoter, respectively, have been described previously [2]. Strain CCP018 was constructed by amplifying a *HOG1* disruption cassette with plasmid pAG25 [3] and oligonucleotides HOG1_NAT_Fw and HOG1_NAT_Rv. The fragment was used to transform BY4741 cells and the deletion confirmed by PCR with the pairs of oligonucleotides HOG1_Comp and K2. Strain CCP020 was made transforming BY4741 cells with a deletion cassette obtained by amplification of the *sko1::kanMX* locus from the corresponding EUROSCARF strain with oligonucleotides SKO1_Fw and SKO1_Rv. The deletion was verified with oligos SKO1_Comp and K2. Strain CCP021 was constructed as strain CCP020 but transforming strain CCP018. Oligonucleotides used in this work are listed in the following table. Strain SP038 was made by transforming strain BY4741 with a *MIG1*::*kanMX4* cassette amplified with oligonucleotides 5'_mig1_disr and 3'_mig1_disr from genomic DNA obtained from the *mig1Δ* EUROSCARF strain. Strain SPO43 was constructed similarly, but transforming strain BY4742 (***MAT*** α) using a *mig2*::*kanM*X4 cassette. Strain SP010 was obtained by crossing strains SP038 and SPO43 and selecting the double mutation by colony PCR of haploid segregants with the BY4741 background.

2. Oligonucleotides used in this work

Name	Sequence	Use
HOG1_NAT_Fw	AATACTAGACTCGAAAAAAAGGAACAAAGGGAAAACAGGGCGTACGCTGCAGGTCGAC	Amplification of natMX6 cassette from pAG25 to construct *hog1* mutant
HOG1_NAT_Rv	TGGTAAGTATACGCTTGTCTGGGTGAGACAGCTATTTAGCATCGATGAATTCGAGCTCG	Amplification of natMX6 cassette from pAG25 to construct *hog1* mutant
HOG1_Comp	AGATACATCCTCAGGGCACC	Primer forward (-720 nt from ATG) to check *hog1*::*nat* integration
SKO1_Fw	CAAGTCGTACTTAACCGCGC	Primer forward (-480 nt from ATG) to amplify *sko1*::*kanMX4* from EUROFAN mutant
SKO1_Rv	TGCGTAATTACGCCCAATGC	Primer reverse (+647 nt after STOP) to amplify *sko1*::*kanMX4* from EUROSCARF mutant
SKO1_Comp	GCGAAAATGGCCAATTTTGG	Primer forward (-874 nt from ATG) to check *sko1*::*kanMX4* integration
5'_mig1_disr	GATGTCTACTCGTCCACATC	Primer forward (-646 nt from ATG) to amplify *mig1*::*kanMX4* integration from EUROSCARF mutant
3'_mig1_disr	GATGTCTACTCGTCCACATC	Primer reverse (+722 nt after STOP) to amplify *mig1*::*kanMX4* from EUROSCARF mutant
5'_mig2_disr	ATTTGAGCTATACCTACCCCA	Primer forward (-646 nt from ATG) to check *mig2*::*kanMX4* integration from EUROSCARF mutant
MIG2-r	CCACCTTATCTCCACGGGAA	Primer reverse (+822 nt after STOP) to amplify *mig2*::*kanMX4* from EUROSCARF mutant
K2	CACGTCAAGACTGTCAAGGA	Verification of *kanMX* and *natMX* deletions
NCE103_prom_5'	GCGAATTCCGCCTGAGCGGCAGTGG	Amplification of the NCE3 promoter
NCE103_prom_3'	CGAAGCTTGTGACTCAATGTGAATATAG	Amplification of the NCE3 promoter

3. Proteomic and phosphoproteomic methods

Extract preparation and protein TMT labeling

To obtain the protein extracts for phosphoproteomic assays, one ml of the chilled (4°C) lysis Buffer C (3% SDS, 0.1 M ammonium bicarbonate, pH 7.5) with the addition of Complete protease inhibitor and PhosStop phosphatase inhibitor mixtures (Roche) was added to the cell pellet together with 200 µl of acid-washed glass-beads (Sigma). The yeast cells were broken in a BeadMill at 4°C with four cycles of 1 min shaking followed by 1 min vortexing. Then, the cell suspension was sonicated in a Bioruptor for 10 min (30 sec sonication followed by 30 sec break cycles) at 4°C, heated at 90°C for 10 min in a water bath and sonicated again in a Bioruptor for 10 additional min. Protein concentration was measured by BCA analysis (Thermo Scientific). The concentration of 100 µg aliquots was adjusted to 1 mg/ml with lysis buffer F, and TCEP (Tris (2-carboxyethyl) phosphine) and CAA (chloroacetamide) were added to 10 mM and 40 mM, respectively. Samples were then heated at 80°C to reduce and alkylate the proteins. Protein material was precipitated by the chloroform/methanol method [4]. Protein pellets were air dried and resuspended in 100 µl of 0.1 M TEAB (Triethylammonium bicarbonate buffer). Trypsin (Promega) was added at a 1/50 ratio and the digestion carried out at 37°C for 12 h. Then, an additional aliquot of trypsin (1/100) was added for 2 h at 37°C in order to improve the digestion.

Peptide solutions were labeled by TMT (Tandem Mass Tag) labelling according to the manufacturer recommendations (TMT11plex Thermo Scientific). The TMT label was dissolved in 50 µl of acetonitrile and mixed with the sample. After 1 h incubation at RT, 8 µl of 5% hydroxylamine were added to quench the reaction. Eighty per cent of the labeled samples were mixed and the relative amount of labeling deduced from the values of the MS2 reporter ion channels. Samples were then rectified, with the objective to equalize the amount of proteins.

Data collection

MS data was collected in DDA mode. For non-modified peptide analysis MS1 parameters were: 120K resolution, 300-1600 scan range, max injection time 15 ms, AGC target $3x10^6$. MS2 scan parameters were: 45K resolution, AGC target $2x10^5$, 80 ms max injection time, NCE 32. Ions were isolated with 1.2 m/z window targeting 15 most intense peaks of +2 to +5 charge with minimum AGC target $2x10^3$. Dynamic exclusion was set to 30 sec. For phosphopeptides MS1 parameters were the same. MS2 parameters were 60K resolution, $1x10^5$ AGC target, 120 ms max injection time, NCE 34. Ions were isolated

with 1.2 m/z window targeting 12 most intense peaks of +2 to +5 charge with minimum AGC target of 1.5x10^3.

References

1. Brachmann CB, Davies A, Cost GJ, Caputo E, Li J, Hieter P, et al. Designer deletion strains derived from Saccharomyces cerevisiae S288C: A useful set of strains and plasmids for PCR-mediated gene disruption and other applications. Yeast. 1998;14: 115–132. doi:10.1002/(SICI)1097-0061(19980130)14:2<115::AID-YEA204>3.0.CO;2-2
2. Calafí C, López-Malo M, Velázquez D, Zhang C, Fernández-Fernández J, Rodríguez-Galán O, et al. Overexpression of budding yeast protein phosphatase Ppz1 impairs translation. Biochim Biophys Acta - Mol Cell Res. 2020;in press.
3. Goldstein AL, McCusker JH. Three new dominant drug resistance cassettes for gene disruption in Saccharomyces cerevisiae. Yeast. 1999;15: 1541–1553.
4. Friedman DB. Quantitative proteomics for two-dimensional gels using difference gel electrophoresis. Methods in molecular biology (Clifton, NJ). 2007. pp. 219–239. doi:10.1385/1-59745-275-0:219

Supplementary Figure S1. a) Transcriptomic data was mapped onto metabolic pathways using YeastPathways at SGD. Pathway Perturbation Score (PPS) measures the overall extent to which a pathway is up- or down-regulated. Differential PPS (DPPS) computes the maximum extent to which a pathway is perturbed between time-points. See main text for details. **b**) *LacZ* reporter assay of four promoters from selected genes (*GAP1*, *GRE2* and *NCE103*, up-regulated, and *SIT1*, down-regulated according to RNA-seq) in BY4741 (empty bars) and ZCZ01 (dark bars) after the addition of galactose. Data is the mean ± SEM from three independent clones. *, *p* <0.05, **, *p*<0.005, ***, *p*<0.001.

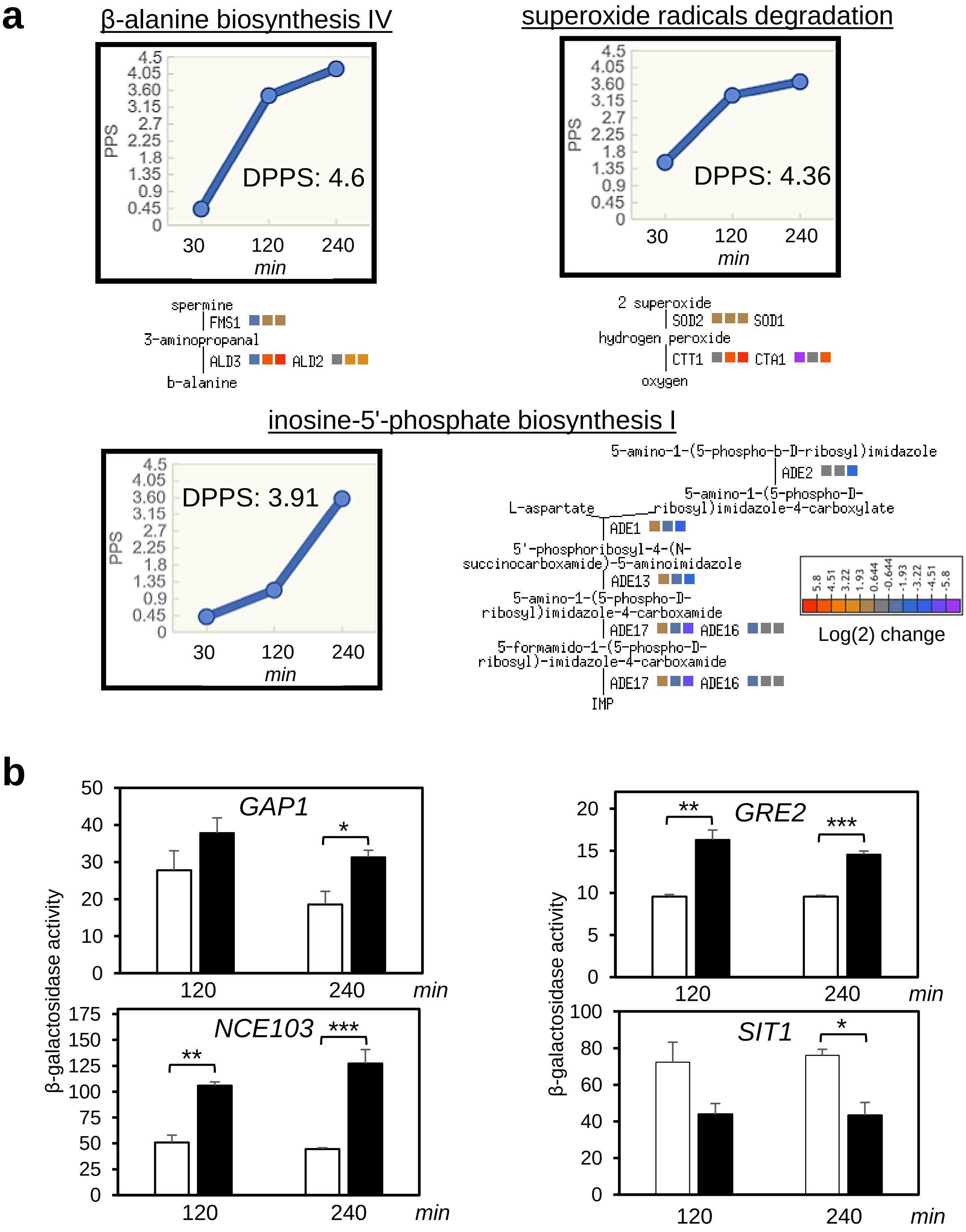

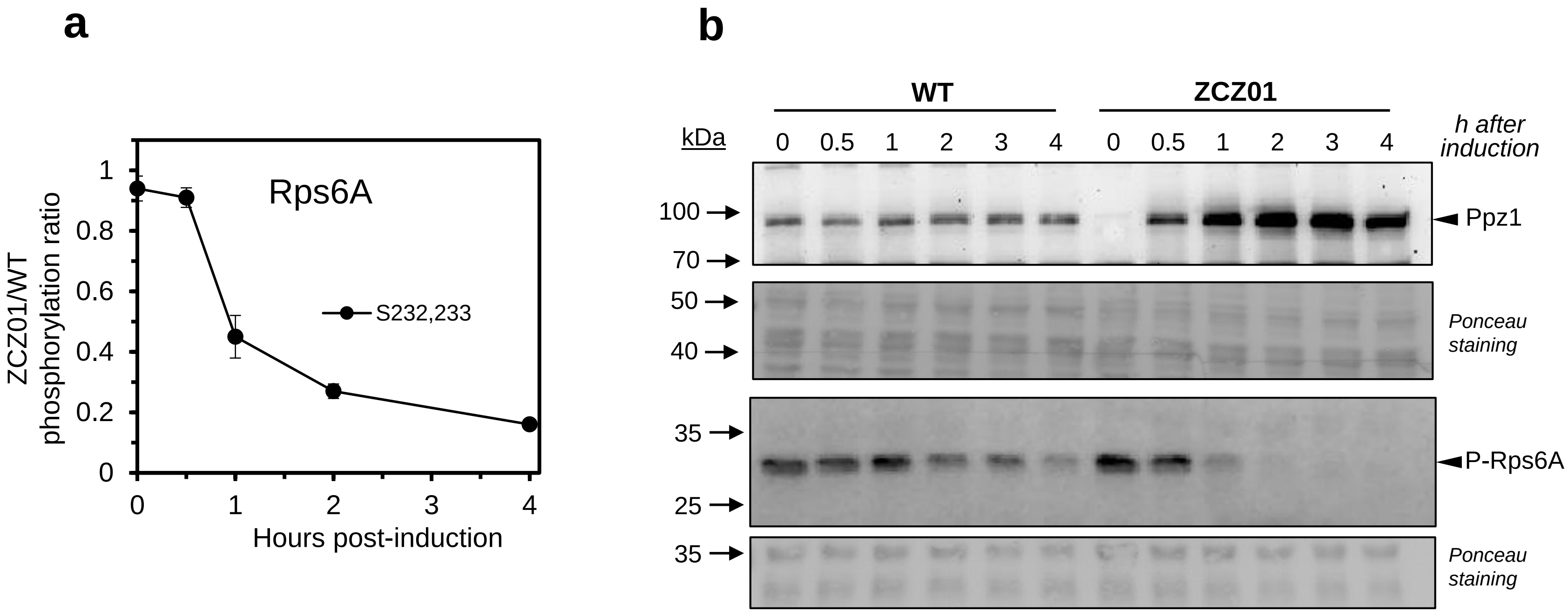

Supplementary Figure S2. a) Changes in phosphorylation in residues S232, 233 of Rps6A from the phosphoproteomic analysis. Data represent the mean ± SEM from 4 experiments. b) BY4741 (WT) and ZCZ01 cells were grown on YP-Raff, induced by addition of galactose (2%), and cells collected at the indicated times. Protein extracts were prepared as described in Materials and Methods and electrophoresed in parallel in 10% gels. Proteins were transferred to membranes and probed with anti-Ppz1 antibodies (upper panels) or anti-Phospho-S6 Ribosomal Protein (Ser235/236) antibodies (bottom panels). Ponceau staining of the membranes is shown to evaluate loading and transfer efficiency.

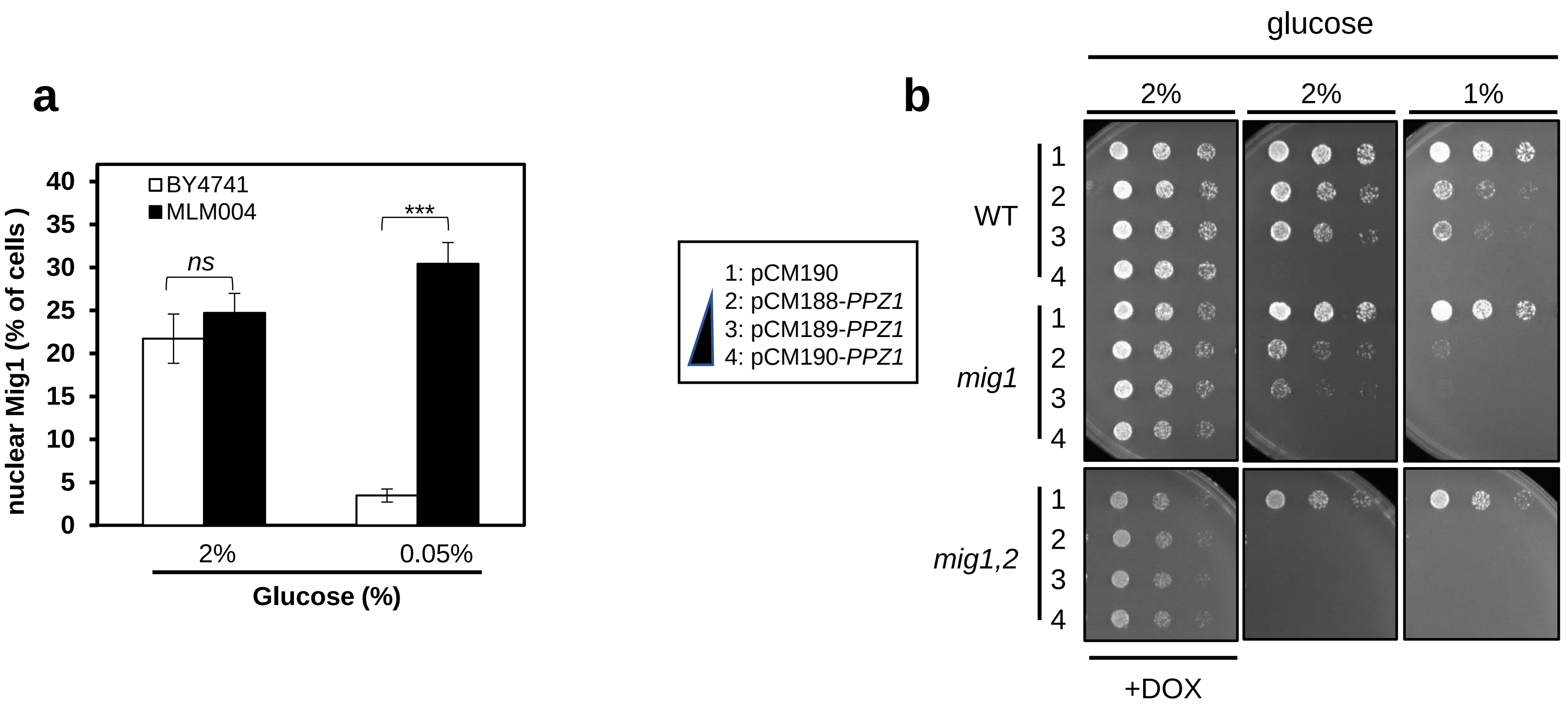

Supplementary Figure S3. a) Nuclear retention of Mig1 in the MLM04 Ppz1-overexpressing strain. BY4741 and MLM04 (*tetO*$_7$.:*PPZ1*) cells were grown overnight in SC medium lacking uracil plus 100 µg/ml of doxycycline. Cells were diluted in the same medium lacking doxycycline and growth resumed for 24 h. Cells were then brought to an OD_{600} of 0.2 and split in two aliquots. One was resuspended in the same medium with 2% glucose and the other with 0.05% glucose. Growth was resumed for 4.5 h and cells were fixed as described in Materials and Methods for microscopic counting. Data are mean ± SEM from three independent experiments (total cells counted 962 to1174). *ns*, not significative (p>0.05); ***, p<0.0005). **b)** BY4741 (WT) and its isogenic derivatives SP038 (*mig1*) and SP010 (*mig1 mig2*) were transformed with the indicated plasmids that different levels of *PPZ1* expression (pCM188 the lowest, pCM190 the highest). Overnight cultures were grown in SC medium lacking uracil with 100 µg/ml of doxycycline, were washed twice with the same medium lacking doxycycline, resuspended in this medium and grown for five hours before spotting in plates with (+ DOX) or without doxycycline and containing the indicated amounts of glucose as carbon source. Pictures were taken after 2 days.

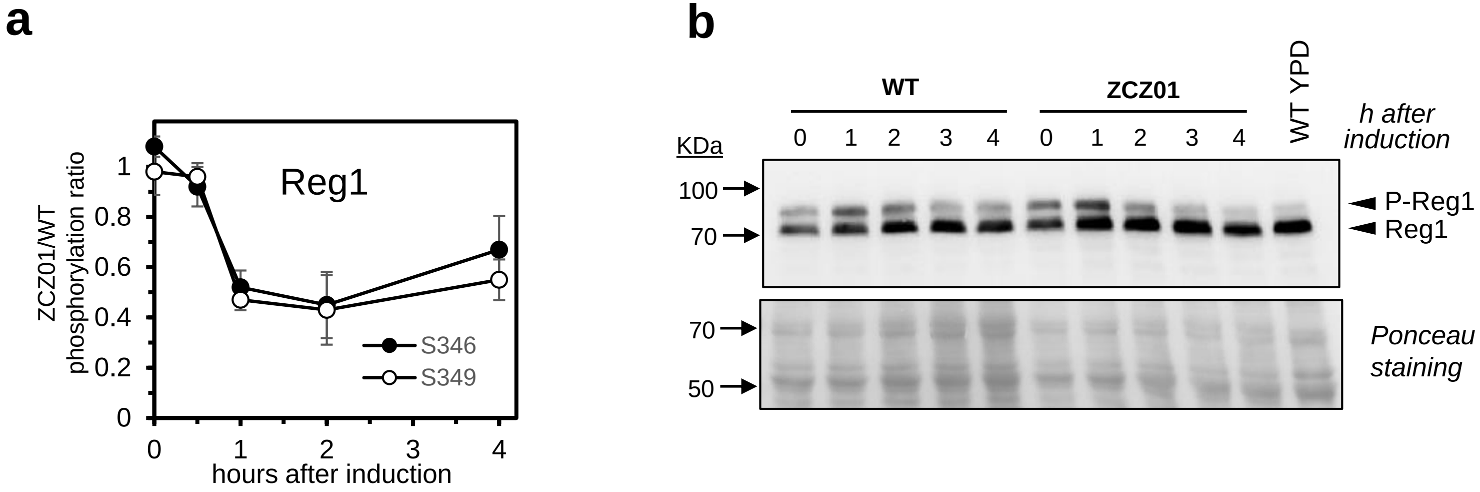

Supplementary Figure S4. **a**) Changes in phosphorylation of Reg1 at residues S346, 349 derived from the phosphoproteomic analysis. Data represent the mean ± SEM from 4 experiments. **b**) BY4741 (WT) and ZCZ01 cells were transformed with plasmid pREG1-HA$^{(1\text{-}443)}$, expressing an HA-tagged version a N-terminal fragment of Reg1. Cultures grown on YP-Raff were induced by addition of galactose (2%), and cells collected at the indicated times. Protein extracts were prepared as described in Materials and Methods and electrophoresed (10% gels). Proteins were transferred to membranes and probed with anti-HA antibodies, followed by anti-mouse IgG secondary antibodies. Bottom panel shows Ponceau staining of the membrane.

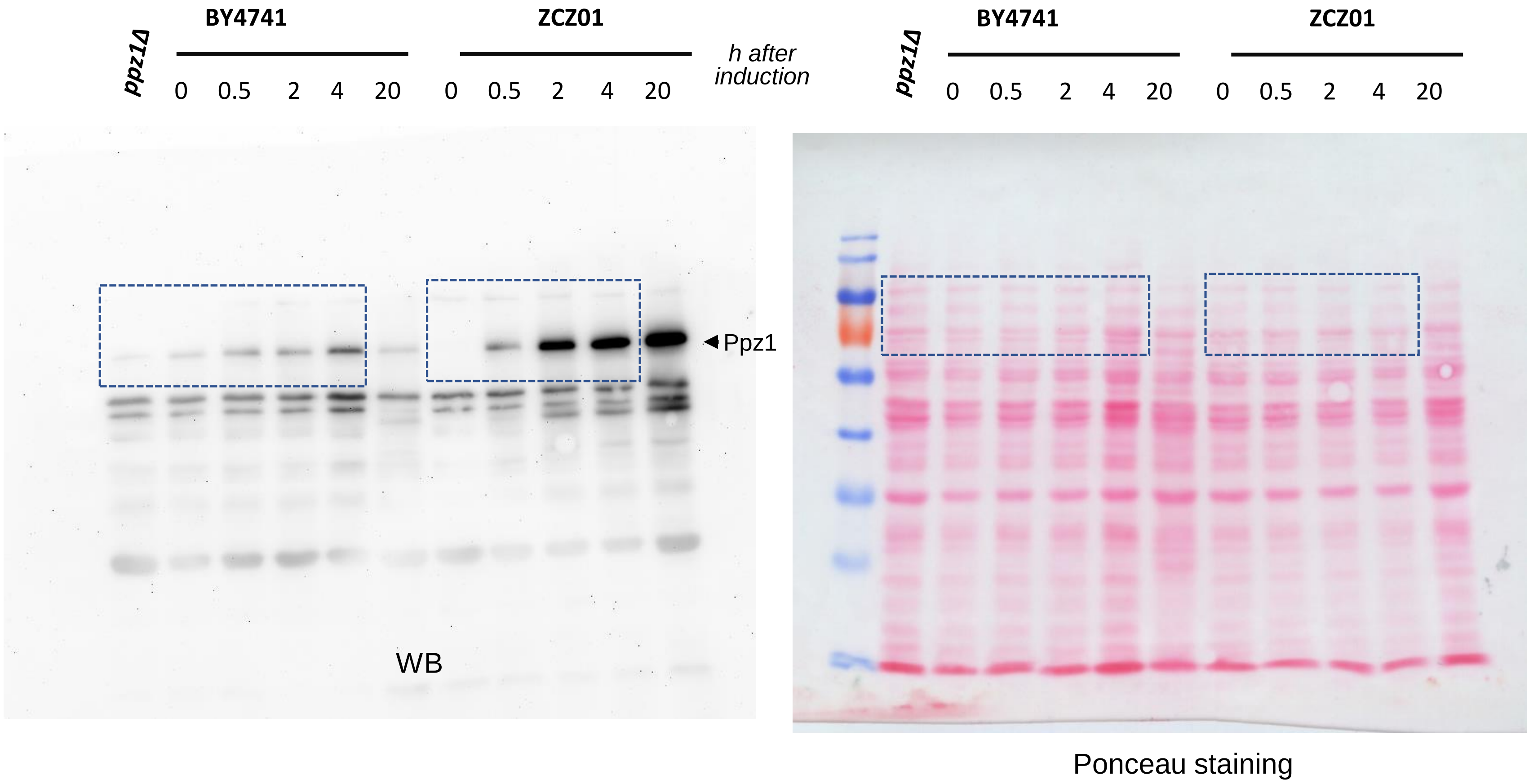

Figure 1a. Full-length gel and immunoblot

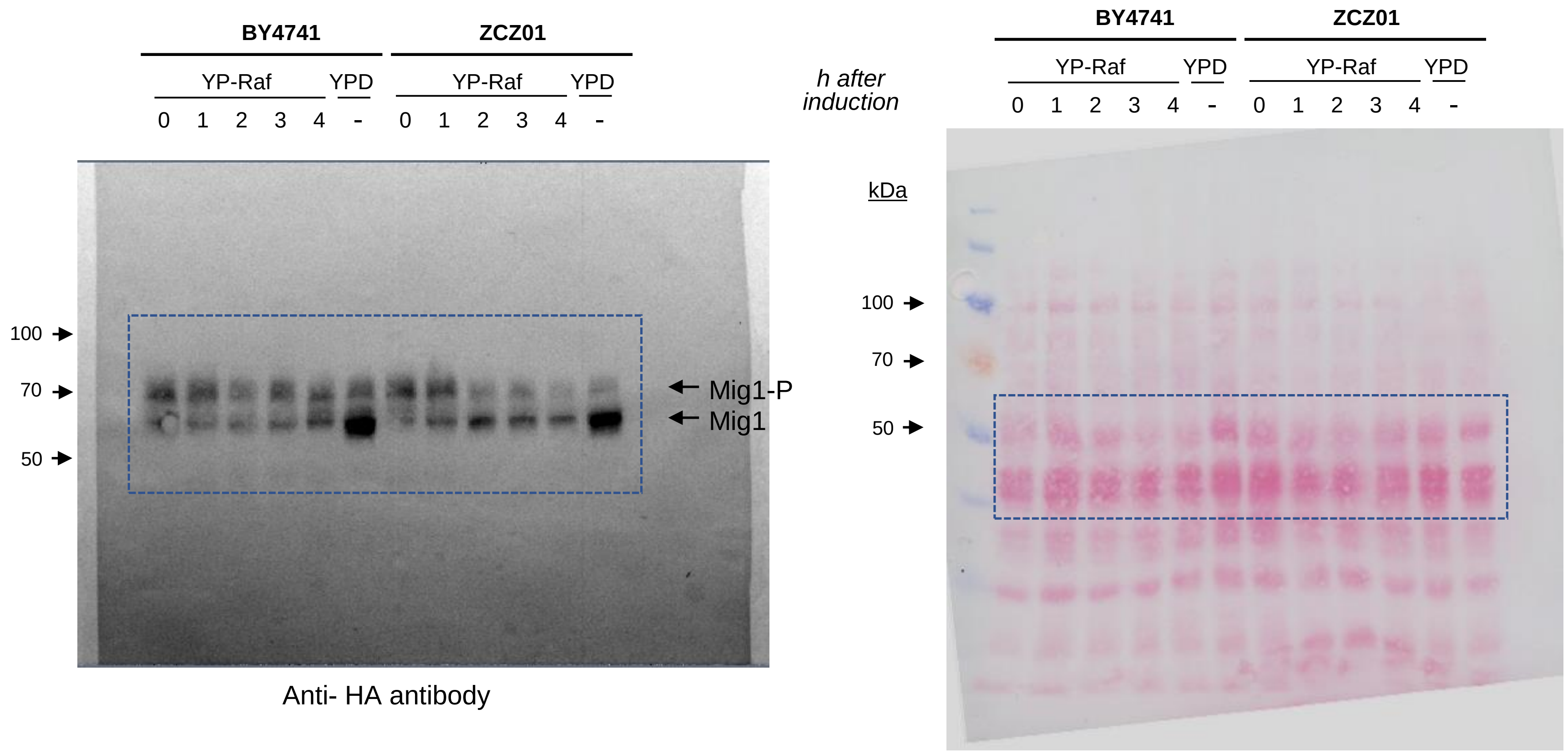

Figure 7a. Full-length gel and immunoblot

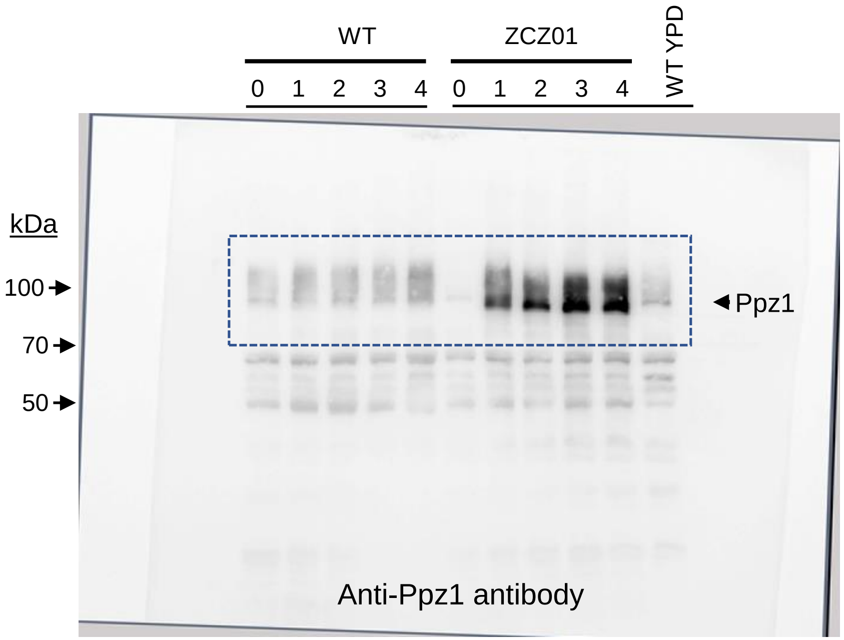

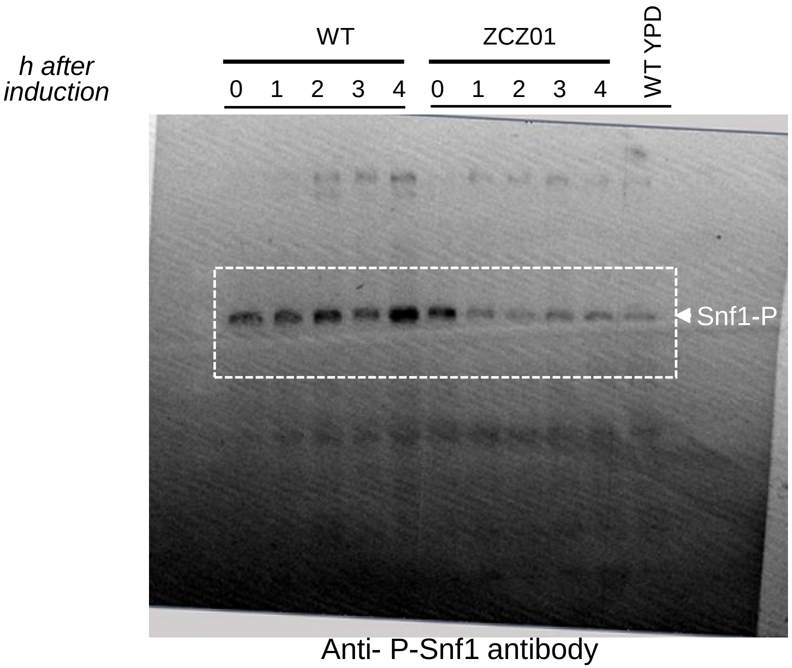

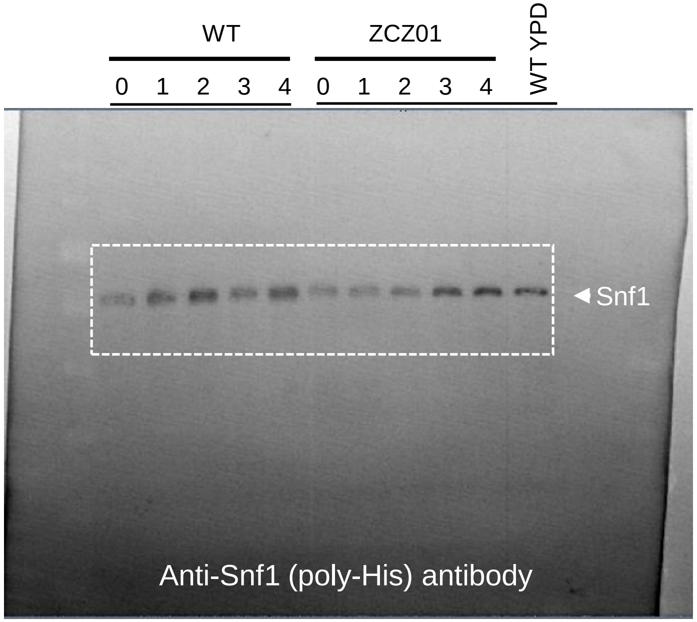

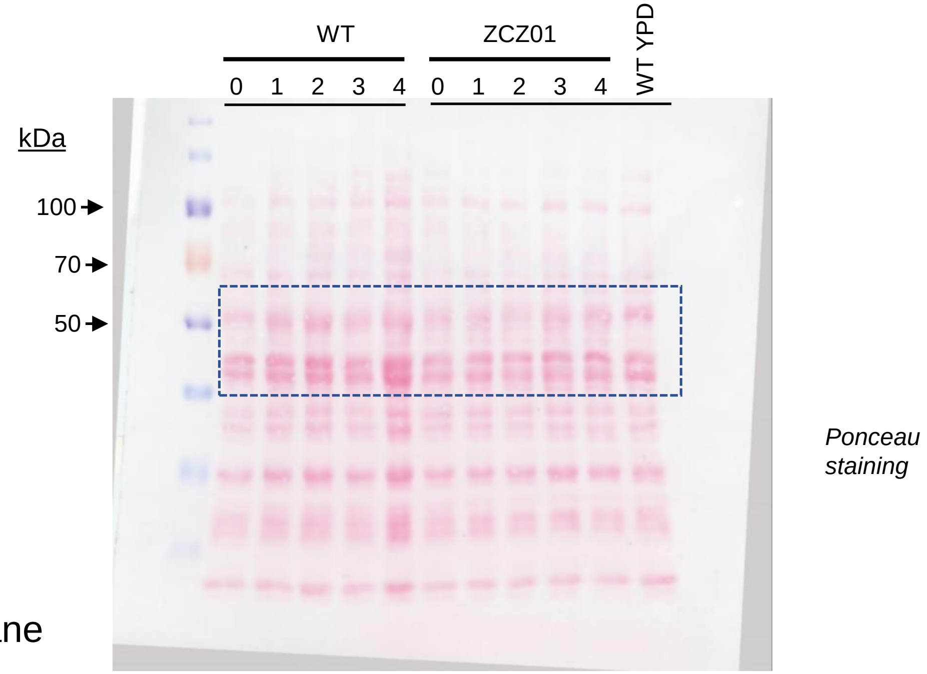

Figure 7c. Full-length gel and blot- reprobed membrane

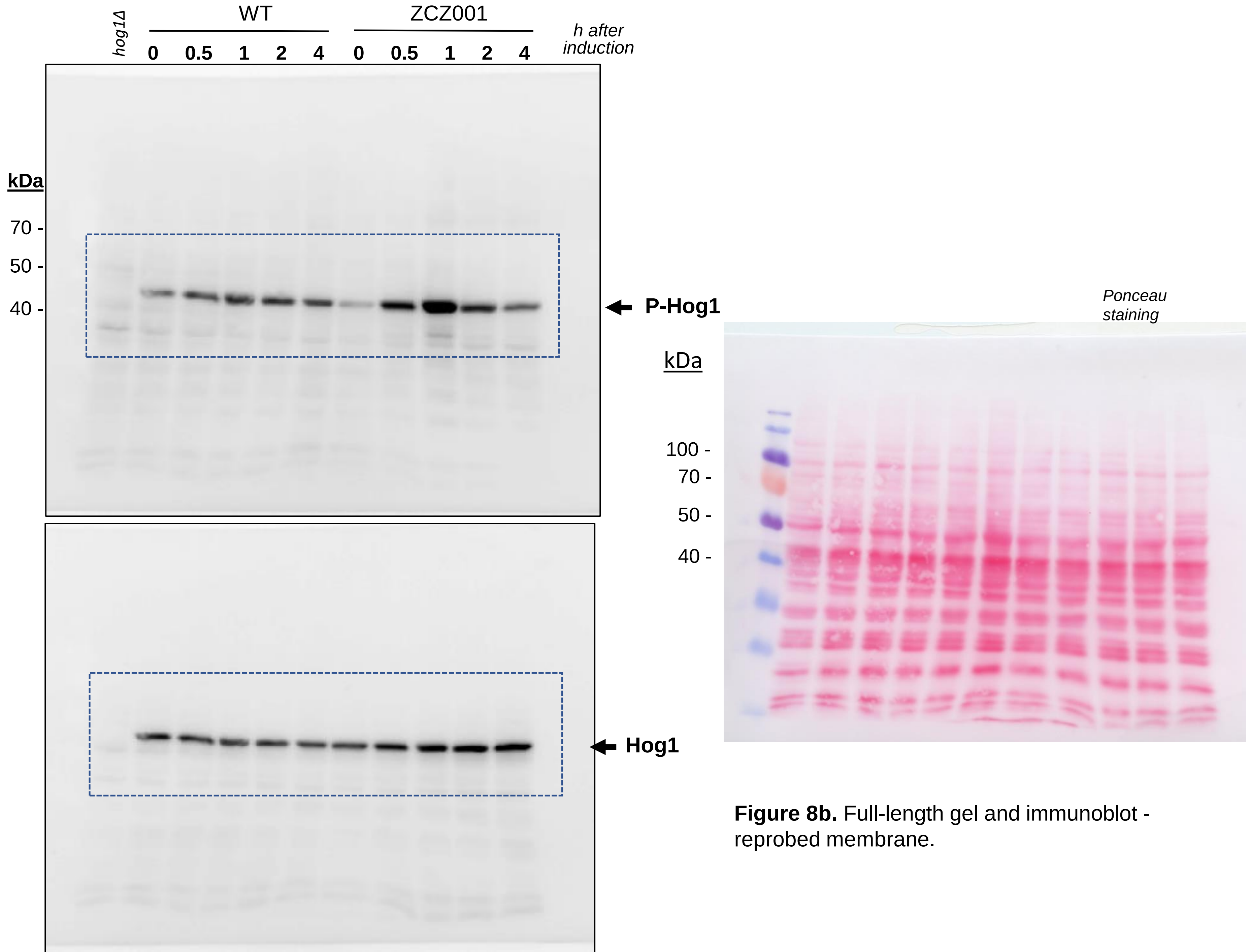

Figure 8b. Full-length gel and immunoblot - reprobed membrane.

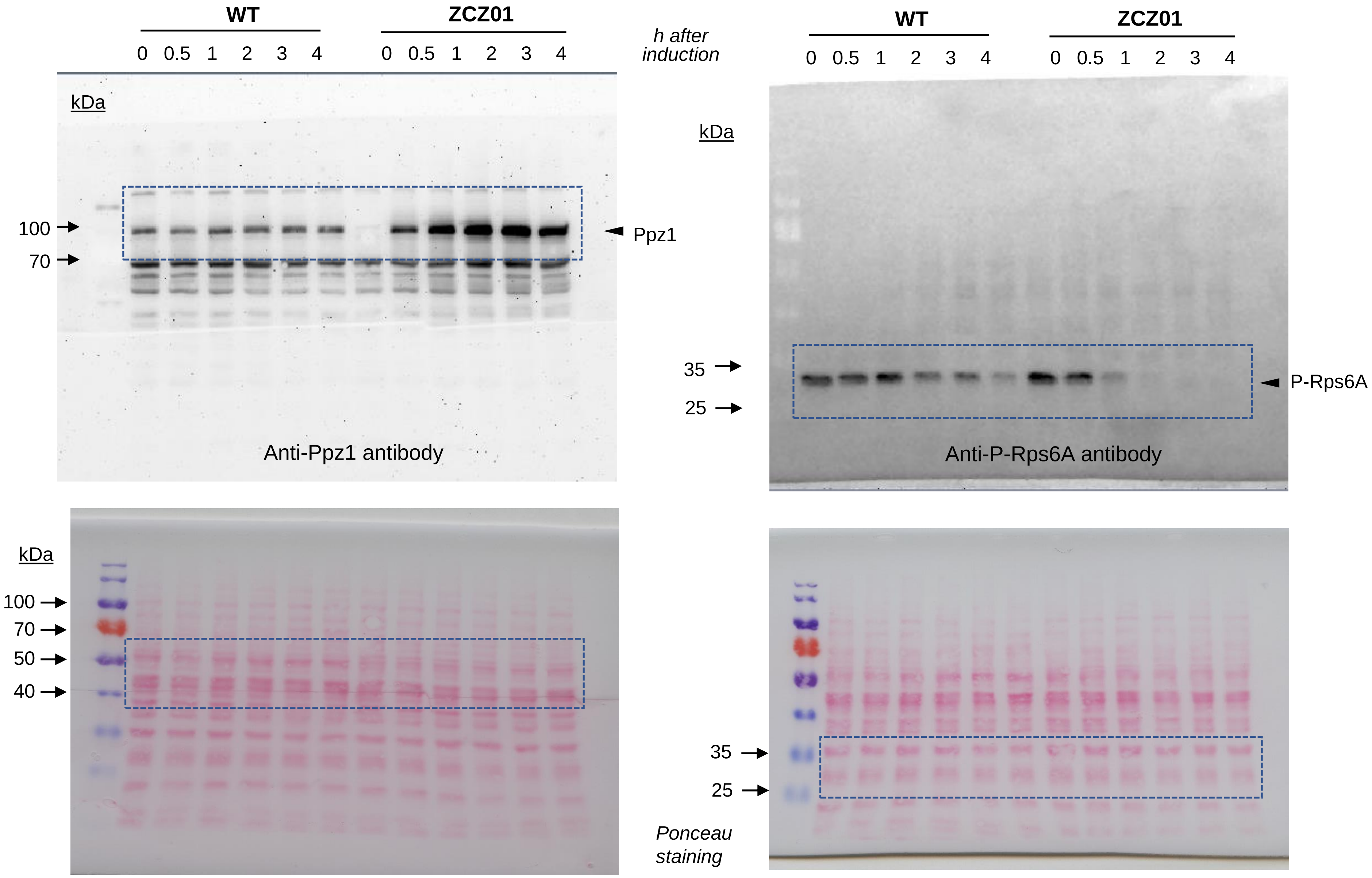

Figure S2. Full-length gels and immunoblots

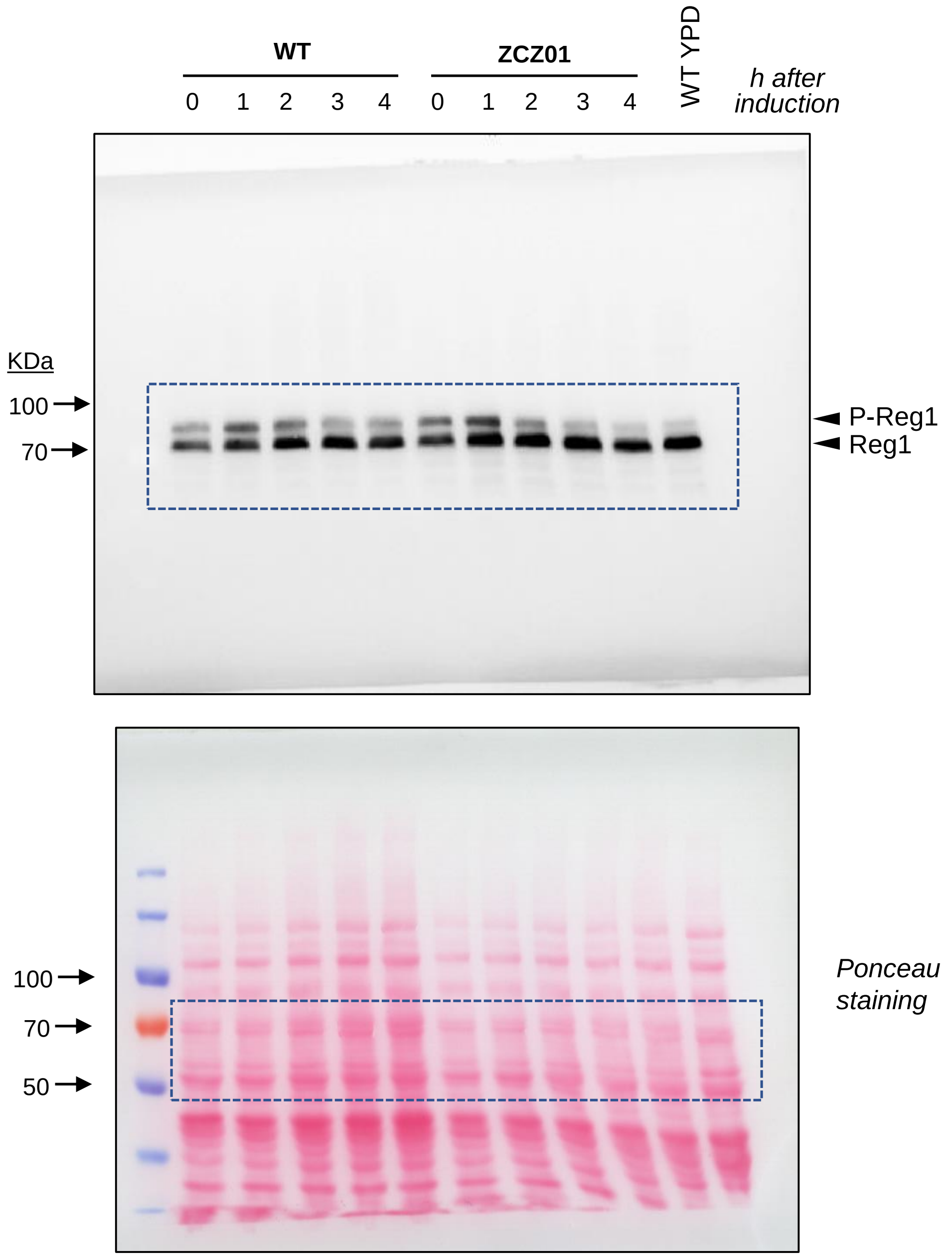

Figure S4. Full-length gels and immunoblots
